# The Set of Serine Peptidases of the *Tenebrio molitor* Beetle: Transcriptomic Analysis on Different Developmental Stages

**DOI:** 10.3390/ijms25115743

**Published:** 2024-05-25

**Authors:** Nikita I. Zhiganov, Konstantin S. Vinokurov, Ruslan S. Salimgareev, Valeriia F. Tereshchenkova, Yakov E. Dunaevsky, Mikhail A. Belozersky, Elena N. Elpidina

**Affiliations:** 1 A.N. Belozersky Institute of Physico-Chemical Biology, Lomonosov Moscow State University, Moscow 119991, Russia; nikitoooc@rambler.ru (N.I.Z.); dun@belozersky.msu.ru (Y.E.D.); mbeloz@belozersky.msu.ru (M.A.B.); 2 Institute of Plant Molecular Biology, Biology Centre of the Czech Academy of Sciences, Branišovská 1160/31, 370 05 České Budejovice, Czech Republic; orchesia@gmail.com; 3 Faculty of Bioengineering and Bioinformatics, Lomonosov Moscow State University, Moscow 119991, Russia; russal2010@fbb.msu.ru; 4 Faculty of Chemistry, Lomonosov Moscow State University, Moscow 119991, Russia; v.tereshchenkova@gmail.com

**Keywords:** *Tenebrio molitor*, serine peptidases, serine peptidase homologs, polypeptidases, phylogenetic analysis, expression patterns, digestion

## Abstract

Serine peptidases (SPs) of the chymotrypsin S1A subfamily are an extensive group of enzymes found in all animal organisms, including insects. Here, we provide analysis of SPs in the yellow mealworm *Tenebrio molitor* transcriptomes and genomes datasets and profile their expression patterns at various stages of ontogeny. A total of 269 SPs were identified, including 137 with conserved catalytic triad residues, while 125 others lacking conservation were proposed as non-active serine peptidase homologs (SPHs). Seven deduced sequences exhibit a complex domain organization with two or three peptidase units (domains), predicted both as active or non-active. The largest group of 84 SPs and 102 SPHs had no regulatory domains in the propeptide, and the majority of them were expressed only in the feeding life stages, larvae and adults, presumably playing an important role in digestion. The remaining 53 SPs and 23 SPHs had different regulatory domains, showed constitutive or upregulated expression at eggs or/and pupae stages, participating in regulation of various physiological processes. The majority of polypeptidases were mainly expressed at the pupal and adult stages. The data obtained expand our knowledge on SPs/SPHs and provide the basis for further studies of the functions of proteins from the S1A subfamily in *T. molitor*.

## 1. Introduction

Serine endopeptidases of the chymotrypsin S1A subfamily are a large group of enzymes widely distributed in nature. In insects, they play an important role in various physiological processes such as digestion, development, and innate immunity regulation [1,2,3,4,5,6,7,8]; therefore, these peptidases are of significant interest for further study. Activity of SPs depends on a catalytic triad of amino acid residues: histidine H57, aspartic acid D102, and serine S195 (hereinafter bovine chymotrypsinogen A numbering, XP_003587247). The substrate specificity of SPs is largely determined by the structure of the S1 substrate-binding subsite, where residues 189, 216, and 226 play the major roles [9]. According to the S1 pocket organization, various types of SPs are distinguished, including trypsins (D189, G216, G226), chymotrypsins (S189, G216, G226; S189, G216, A226, and others), and elastases (S189, V216, T226; S189, V216, D226, and others).

**Figure 1:**
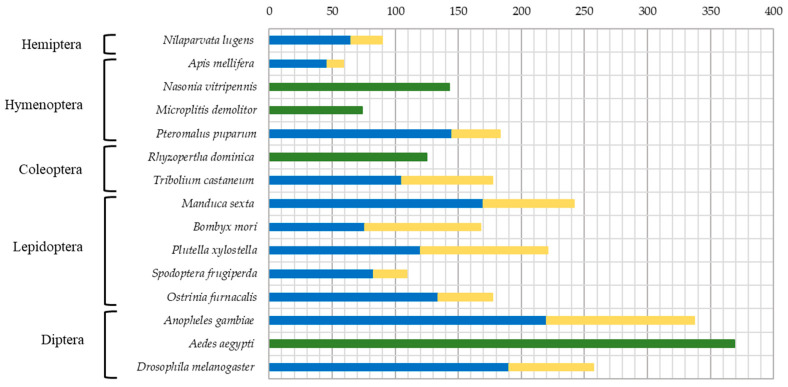
Total number of SP and SPH genes found in sequenced genomes of insects from different orders. Data on SP are shaded in blue, data on SPH are in yellow, and undifferentiated data on the sum of SP/SPH genes are shaded in green.

Development of high throughput sequencing technologies lead to the appearance of high-quality genome assemblies for the whole-genome investigation of SP/SPH genes, performed for model insects, as well as species of great agricultural and medical importance. Among Hemiptera, 90 SP/SPH genes were found in *Nilaparvata lugens* (Delphacidae) [10] (Figure 1). In Dipterans, 257 genes were identified in *Drosophila melanogaster* (family Drosophilidae) [11,12] and even more in mosquitoes (family Culicidae) *Anopheles gambiae*—337 [13,14], and *Aedes aegypti*—369 [15,16]. For the order Lepidoptera, data on several representatives are known: 242 genes were found in *Manduca sexta* (Sphingidae) [17,18], 169 genes in *Bombyx mori* (Bombycidae) [19,20], 221 genes in *Plutella xylostella* (Plutellidae) [21], and 109 genes in *Spodoptera frugiperda* (Noctuidae) [22]. A reduced set of only 57 SP/SPH genes was found in *Apis mellifera* (Hymenoptera: Apidae) [12,23]. The gene repertoire was larger in parasitic Hymenoptera with 74 genes described in *Microplitis demolitor* (Braconidae), 143 genes in the parasitic wasp *Nasonia vitripennis*, and 183 genes in *Pteromalus puparum* (Pteromalidae) [24].

Genome-wide analyses in beetles (Coleoptera) identified 125 SP/SPH genes in *Rhyzopertha dominica* (Bostrichidae) [25]. From the first coleopteran sequenced genome of the red flour beetle *Tribolium castaneum* (Tenebrionidae), 177 genes coding for SPs/SPHs were identified [12,26]. For another tenebrionid, the yellow mealworm *Tenebrio molitor*, 38 SP/SPH transcripts were previously identified in the larval gut [27], two of which corresponded to the major digestive trypsin and chymotrypsin studied using biochemical approaches [28,29]. Later, 48 SP/SPH transcripts were identified in the larval gut during the study of Cry3A intoxication in *T. molitor* [30]. Analyzing trypsin-like SPs/SPHs in transcriptome datasets from different stages of the *T. molitor* life cycle, we have previously de novo assembled 54 trypsins and five trypsin-like SPHs [31]. We also characterized recombinant preparations of SP, SerP38, and SPH, SerPH122, expressed in the *Komagataella kurtzmanii* system [32,33]. Recent work by Wu and coauthors [34] provided information on 200 *T. molitor* genes including 112 SPs and 88 SPHs, and transcriptome datasets together with RT-PCR analysis were used for SP-related genes expression profiling at various developmental stages and tissues.

Here, we present the extended and corrected dataset of putative *T. molitor* SP/SPH cDNAs obtained from genome and transcriptome datasets. We have identified several groups of deduced proteins based on the composition of their active site and predicted specificity, analyzed evolutionary relationships and evaluated differential expression along the life cycle. Finally, sets of SP-related genes involved in digestion, embryonic development, metamorphosis, and innate immunity were predicted, providing valuable information for further physiological, biochemical, and phylogenetic studies of tenebrionid pests. These data are of particular interest due to the fact that *T. molitor* is the first insect approved by the European Food Safety Authority as a novel food in specific conditions and uses, testifying its growing relevance and potential [35].

## 2. Results

### 2.1. General Characteristics of T. molitor Predicted SPs/SPHs of the S1A Subfamily

#### 2.1.1. Identified Set of Peptidase-like Sequences

Analysis of the total *T. molitor* transcriptome assembly, transcriptomes from different developmental stages coupled with verification of sequences in three new whole genome assemblies (GCA_027725215.1; GCA_014282415.3; GCA_907166875.3), revealed a total of 269 mRNA sequences encoding putative SPs and SPHs. Of these, 137 were transcripts of active SPs with a conserved catalytic triad of amino acid residues in the active center—H57, D102, S195, whereas 125 sequences having one or more substitutions in the catalytic triad were SPHs. In addition, there were seven sequences of polypeptidases (polyserases in humans according to [36]) containing two or three tandem peptidase domains, SP and/or SPH, translated from a single ORF as an integral part of the same polypeptide chain. 

Bioinformatics analysis allowed us to discover 69 new sequences, and the structure of another 23 sequences previously available [27,31,34] was revised and reannotated.

#### 2.1.2. Annotation of Predicted Protein Sequences of *T. molitor* SPs

The sequences of active SPs were analyzed by the composition of the S1 substrate-binding subsite, where three amino acid residues in positions 189, 216, and 226 reflect to a large extent the specificity of the peptidase [9]. We identified trypsins as SPs with a conserved set of amino acid residues in the S1 subsite—D189, G216, G226 (DGG), bringing the negative charge to the S1 pocket base, ensuring specificity for basic residues (R/K) at the P1 position of the substrate [37]. Those with A, T, or S at positions 216 or 226 instead of G, while keeping negatively-charged D at the bottom (DGA; DGT; DSG; DAT), were tentatively named as trypsin-like, although their specificity is questionable due to larger side chains located at the pocket walls. Predicted peptidases lacking the negative charge at the base of the S1 pocket were defined as chymotrypsin- or elastase-like according to the residues that occupy the wall positions 216 and 226. Those with small amino acid residues (SGS; SGA; GGS; GAS; GSG; SSG) including sequences with negative charge in the pocket wall (GGD), characteristic of insects [38], were predicted as chymotrypsin-like, for which specificity towards large aromatic (F, Y, W) or mid-size aliphatic (L) side chains in the P1 position is generally accepted. Whereas in putative elastase-like SPs, wall position 216 occupied by bulky hydrophobic residues (SVS; GVS; GVN; GIS; GFS; GYS) generally provides a platform for interaction with small hydrophobic residues at P1. A group of non-annotated peptidases with an unusual S1 subsite was also established, for which specificity could not be reasonably predicted from sequence analysis. The most numerous SPs were trypsins with 64 sequences. Other groups included 10 trypsin-like peptidases, 30 chymotrypsin-like peptidases, 18 elastase-like and 15 non-annotated peptidases.

#### 2.1.3. Domain Organization

To propose the functional role of *T. molitor* SPs/SPHs, their domain organization was studied. The vast majority of the sequences were presented as preproenzymes. The predomain or N-terminal signal peptide responsible for the secretory pathway was found in 262 sequences out of 269 studied. Eighty-three sequences contained one or more regulatory domains in the propeptide structure responsible for various physiological functions in the insect. Namely, these were 53 sequences out of 137 SPs with the classical catalytic triad, 23 sequences out of 125 SPHs, and all sequences of polypeptidases contained regulatory domains. Thirteen peptidases had a transmembrane domain (TM). Among them, seven had a TM at the N-terminus and six at the C-terminus. Most sequences of mature enzymes without prodomain contained 225–260 amino acid residues.

### 2.2. Trypsins and Trypsin-like Peptidases

**Figure 2:**
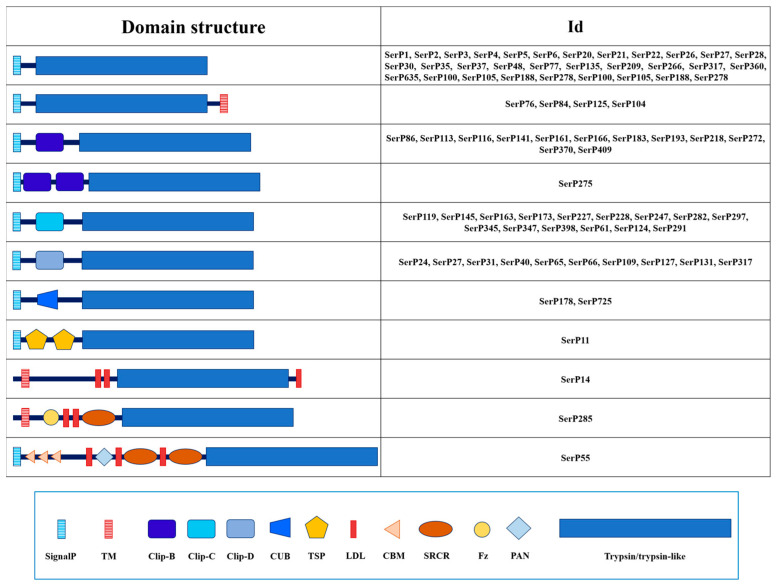
Domain organization of 64 trypsins and 10 trypsin-like SPs of *T. molitor*. Regulatory domains are marked with different shapes and colors. Description for domains: SignalP—signal peptide; TM—transmembrane domain; Clip (B/C/D)—Clip domain; CUB—C1r/C1s, Uegf, Bmp1 domain; TSP—thrombospondin domain; LDL—Low-Density Lipoprotein receptor class A repeat; CBM—Chitin-Binding Domain; SRCR—Scavenger Receptor Cysteine-Rich domain; Fz—Frizzled domain; PAN—Plasminogen-Apple-Nematode domain.

In *T. molitor* transcriptome dataset, transcripts coding for putative trypsin-related proteins constituted the most numerous group: 64 trypsin sequences and 10 trypsin-like. Sequence analysis revealed that 39 trypsins were mosaic containing a variety of non-catalytic regulatory domains in the propeptide, as well as 6 trypsin-like sequences, and only 25 trypsins and 4 trypsin-like peptidases had no regulatory regions in the propeptide, but 4 trypsins had a transmembrane region in the C-terminal end of the sequence (Table 1, Figure 2).

Most of SPs without regulatory domains are probably activated by trypsins, since 24 out of 25 sequences demonstrate conserved cleavage (activation) site with R or K residues at the carboxyl side of the scissile bond (P1) and hydrophobic branched V or I at the P1′, indispensable for stabilization of new active conformation by hydrogen bonding to D194, the preceding residue to the catalytic S195 [39]. Non-tryptic activation (processing) of the proenzyme is proposed for only single trypsin SerP135 with G residue at P1 of the scissile bond, and single trypsin-like SerP105 with L residue at P1, both from the group of SPs without regulatory domains. In the group of trypsins and trypsin-like *T. molitor* SPs with regulatory domains, 16 sequences have mainly hydrophobic residues at the C-terminal of the propeptide, which do not match the specificity of trypsin and are presumably activated by other peptidases. It should be noted that none of the *T. molitor* trypsins compared to its mammalian counterparts contain a consensus motif for recognition and cleavage by enteropeptidase (DDDDK#) [40], suggesting an alternative regulation of zymogens conversion into active enzymes in insect midgut lumen. 

Among the 45 mosaic sequences with one or more regulatory regions in the propeptide, clip domains of several different types represent the most abundant non-catalytic structural unit of these trypsin-related proteins. A total of 35 clip domain trypsins were identified, including 12 with clip-B, 12 with clip-C, and 11 with clip-D type domains, revealed according to the classification provided earlier [41]. Among 10 sequences of trypsin-like peptidases, which had substitutions in the structure of the S1 subsite (7 with DGA, and single DAT, DGT, and DSG) (Table 1), 4 of 6 sequences with regulatory regions had clip domains (1 with clip-B and 3 with clip-C) and 2 had the CUB domain (CUB, IPR000859) (Figure 2). The remaining four mosaic sequences of true trypsins contained chitin-binding modules (CBM, IPR002557), low-density lipoprotein receptor type A repeats (LDL, IPR002172), scavenger receptor cysteine-rich domain (SRCR, IPR017448), thrombospondin type 1 repeats (TSP, IPR000884), Frizzled domain (Fz, IPR020067), Pan/Apple domain (PAN, IPR003609), and a domain in Complement 1r/s, Uegf and Bmp1 (CUB, IPR000859). 

The isoelectric point (pI) of true trypsins and trypsin-like SPs varied over a wide pH range from 4.3 to 9.5 pH units, suggesting possible involvement of these SPs in different physiological processes. 

**Table 1:** Domain organization and key structure features of 64 trypsins and 10 trypsin-like SPs of *T. molitor*.

№	Name	NCBI ID (Protein)	Preproenzyme/Mature Enzyme (aa)		SignalP (aa)	Regulatory Domain	Propeptide Cleavage Site	Active Site			S1 Subsite			Enzyme Specificity	Mm Mature, Da	pI	TM (Position)
1	SerP1	ABC88729	258	227	16	-	**R**|IVGG	H	D	S	D	G	G	Trypsin	22,742	6.9	-
2	SerP2	QWS65012	252	227	16	-	**R**|IVGG	H	D	S	D	G	G	Trypsin	23,618	4.3	-
3	SerP3	QWS65044	259	228	16	-	**K**|IVGG	H	D	S	D	G	G	Trypsin	24,386	5.0	-
4	SerP4	QWS65013	250	225	15	-	**R**|IVGG	H	D	S	D	G	G	Trypsin	24,140	5.2	-
5	SerP5	QWS65045	333	236	24	-	**R**|IVGG	H	D	S	D	G	G	Trypsin	26,035	9.2	-
6	SerP6	QWS65014	258	226	17	-	**R**|IVGG	H	D	S	D	G	G	Trypsin	23,414	3.8	-
7	SerP20	QWS65048	361	238	17	-	**R**|IVGG	H	D	S	D	G	G	Trypsin	26,395	9.0	-
8	SerP21	QWS65049	276	228	22	-	**R**|IVGG	H	D	S	D	G	G	Trypsin	24,732	4.5	-
9	SerP22	QWS65050	290	242	17	-	**R**|VVGG	H	D	S	D	G	G	Trypsin	25,975	6.2	-
10	SerP26	QWS65055	254	227	23	-	**R**|IVGG	H	D	S	D	G	G	Trypsin	24,214	5.8	-
11	SerP28	QWS65056	310	241	26	-	**R**|IVGG	H	D	S	D	G	G	Trypsin	27,033	7.6	-
12	SerP30	QWS65015	249	226	16	-	**K**|IIGG	H	D	S	D	G	G	Trypsin	24,862	8.9	-
13	SerP35	QWS65057	260	231	21	-	**R**|IVGG	H	D	S	D	G	G	Trypsin	24,884	5.6	-
14	SerP37	QWS65058	298	251	19	-	**R**|VVGG	H	D	S	D	G	G	Trypsin	27,327	6.2	-
15	SerP48	QWS65017	321	295	22	-	**R**|IVGG	H	D	S	D	G	G	Trypsin	32,018	6.7	-
16	SerP76	QWS65019	387	362	18	-	**K**|IIGG	H	D	S	D	G	G	Trypsin	39,417	5.7	367–386
17	SerP77	QWS65060	288	252	17	-	**K**|IVGG	H	D	S	D	G	G	Trypsin	27,164	8.3	-
18	SerP84	QWS65020	332	308	20	-	**K**|VVGG	H	D	S	D	G	G	Trypsin	33,286	5.0	313–330
19	SerP104	QWS65061	323	300	18	-	**K**|IVGG	H	D	S	D	G	G	Trypsin	32,646	4.2	300–323
20	SerP125	QWS65024	278	254	19	-	**R**|IVGG	H	D	S	D	G	G	Trypsin	27,535	4.8	257–275
21	SerP135	QWS65027	292	246	22	-	**G**|IIGG	H	D	S	D	G	G	Trypsin	26,850	9.5	-
22	SerP209	QWS65033	258	227	16	-	**R**|IIGG	H	D	S	D	G	G	Trypsin	22,943	4.8	-
23	SerP266	QWS65037	281	256	18	-	**K**|IVGG	H	D	S	D	G	G	Trypsin	27,895	8.8	-
24	SerP360	CAH1374004	286	249	19	-	**K**|IVGG	H	D	S	D	G	G	Trypsin	27,480	4.7	-
25	SerP635	WJL97986	249	224	19	-	**R**|IVGG	H	D	S	D	G	G	Trypsin	24,044	4.1	-
26	SerP100	WJL97987	293	263	23	-	**R**|IIGG	H	D	S	D	G	A	Trypsin-like	28,605	8.8	-
27	SerP105	CAH1374591	305	243	23	-	**L**|IIGG	H	D	S	D	G	A	Trypsin-like	26,155	5.9	-
28	SerP188	KAJ3637256	303	271	20	-	**R**|IVGG	H	D	S	D	G	A	Trypsin-like	29,751	8.3	-
29	SerP278	CAH1363947	298	256	18	-	**R**|IIGG	H	D	S	D	G	A	Trypsin-like	27,716	6.8	-
30	SerP86	QWS65021	458	258	22	Clip-B	**R**|ILDG	H	D	S	D	G	G	Trypsin	28,226	8.4	-
31	SerP113	QWS65022	386	255	23	Clip-B	**R**|IING	H	D	S	D	G	G	Trypsin	28,255	7.7	-
32	SerP116	QWS65063	381	257	16	Clip-B	**K**|IVNG	H	D	S	D	G	G	Trypsin	28,382	6.4	-
33	SerP141	QWS65028	435	259	21	Clip-B	**R**|IFGG	H	D	S	D	G	G	Trypsin	28,844	9.2	-
34	SerP161	WJL97988	278	254	20	Clip-B	**R**|ITSG	H	D	S	D	G	G	Trypsin	27,807	7.7	-
35	SerP166	QWS65064	376	259	15	Clip-B	**K**|LVND	H	D	S	D	G	G	Trypsin	28,449	4.8	-
36	SerP183 SPE	BAG14262	383	265	18	Clip-B	**R**|IYGG	H	D	S	D	G	G	Trypsin	29,203	7.6	-
37	SerP193	QWS65032	375	247	22	Clip-B	**R**|ILGG	H	D	S	D	G	G	Trypsin	27,564	6.2	-
38	SerP272	QWS65038	404	297	17	Clip-B	**K**|IYGG	H	D	S	D	G	G	Trypsin	32,710	8.0	-
39	SerP275	QWS65065	430	257	23	Clip-B (2)	**K**|IVGG	H	D	S	D	G	G	Trypsin	28,969	8.5	-
40	SerP370	QWS65041	407	257	21	Clip-B	**K**|ISNG	H	D	S	D	G	G	Trypsin	28,048	6.4	-
41	SerP409	QWS65042	447	234	22	Clip-B	**K**|IGKG	H	D	S	D	G	G	Trypsin	26,142	8.8	-
42	SerP218	CAH1363991	356	263	22	Clip-B	**K**|VSGG	H	D	S	D	A	T	Trypsin-like	29,129	6.3	-
43	SerP119	QWS65023	387	253	19	Clip-C	**L**|IVGG	H	D	S	D	G	G	Trypsin	28,333	8.1	-
44	SerP145	QWS65029	370	241	22	Clip-C	**H**|IVGG	H	D	S	D	G	G	Trypsin	26,781	7.7	-
45	SerP163	QWS65030	354	254	21	Clip-C	**V**|IAFG	H	D	S	D	G	G	Trypsin	28,041	5.7	-
46	SerP173	QWS65031	362	249	21	Clip-C	**F**|VFGG	H	D	S	D	G	G	Trypsin	27,495	4.9	-
47	SerP227	QWS65034	376	251	23	Clip-C	**L**|IVGG	H	D	S	D	G	G	Trypsin	27,969	5.8	-
48	SerP228 SAE	QWS65035	374	250	20	Clip-C	**L**|IVGG	H	D	S	D	G	G	Trypsin	27,849	6.2	-
49	SerP247	QWS65036	379	257	18	Clip-C	**T**|IISM	H	D	S	D	G	G	Trypsin	28,343	6.1	-
50	SerP282	QWS65039	349	270	17	Clip-C	**G**|ITGG	H	D	S	D	G	G	Trypsin	29,212	6.0	-
51	SerP297	QWS65066	350	255	18	Clip-C	**V**|EYEE	H	D	S	D	G	G	Trypsin	28,238	5.7	-
52	SerP345	QWS65040	359	234	22	Clip-C	**L**|IVGG	H	D	S	D	G	G	Trypsin	26,360	6.5	-
53	SerP347	QWS65067	367	256	25	Clip-C	**G**|IAIG	H	D	S	D	G	G	Trypsin	28,001	5.8	-
54	SerP398	CAH1365893	385	253	19	Clip-C	**L**|IIGG	H	D	S	D	G	G	Trypsin	28,360	8.9	-
55	SerP61	CAH1377522	422	246	26	Clip-C	**L**|IVGG	H	D	S	D	G	A	Trypsin-like	27,368	8.7	-
56	SerP124	CAH1383174	371	250	20	Clip-C	**L**|IVGG	H	D	S	D	S	G	Trypsin-like	27,690	6.0	-
57	SerP291	WJL97989	357	251	20	Clip-C	**Q**|IWGG	H	D	S	D	G	T	Trypsin-like	28,108	7.1	-
58	SerP15	QWS65047	516	235	23	Clip-D	**R**|IVGG	H	D	S	D	G	G	Trypsin	25,699	9.2	-
59	SerP24	QWS65051	810	243	19	Clip-D	**R**|IVGG	H	D	S	D	G	G	Trypsin	27,555	5.4	-
60	SerP27	QWS65052	369	242	19	Clip-D	**R**|IVGG	H	D	S	D	G	G	Trypsin	26,704	9.0	-
61	SerP31	CAH1379474	557	244	15	Clip-D	**K**|IVGG	H	D	S	D	G	G	Trypsin	26,888	6.5	-
62	SerP40	QWS65016	392	241	21	Clip-D	**G**|NPGG	H	D	S	D	G	G	Trypsin	26,535	5.5	-
63	SerP65	QWS65053	619	240	20	Clip-D	**R**|IVGG	H	D	S	D	G	G	Trypsin	26,001	9.2	-
64	SerP66	QWS65059	523	245	29	Clip-D	**R**|VVGG	H	D	S	D	G	G	Trypsin	27,560	9.1	-
65	SerP109	QWS65062	964	247	17	Clip-D	**R**|IVGG	H	D	S	D	G	G	Trypsin	26,987	7.8	-
66	SerP127	QWS65025	376	247	22	Clip-D	**R**|IVNG	H	D	S	D	G	G	Trypsin	27,075	7.0	-
67	SerP131	QWS65026	375	247	22	Clip-D	**R**|VVNG	H	D	S	D	G	G	Trypsin	26,799	8.4	-
68	SerP317	QWS65054	389	246	16	Clip-D	**R**|IIGG	H	D	S	D	G	G	Trypsin	27,195	6.2	-
69	SerP178	KAJ3638924	409	242	27	CUB	**R**|IVGG	H	D	S	D	G	A	Trypsin-like	26,019	5.0	-
70	SerP725	KAJ3638922	405	246	23	CUB	**K**|IVGG	H	D	S	D	G	A	Trypsin-like	26,660	4.9	-
71	SerP285 Corin	CAH1378270	965	247	-	Fz, LDL (2), SRCR	**R**|IVGG	H	D	S	D	G	G	Trypsin	27,268	5.9	338–359
72	SerP14	QWS65046	1289	286	-	LDL (3)	**R**|IVGG	H	D	S	D	G	G	Trypsin	31,448	5.9	68–94
73	SerP11 TSP	QWS65043	447	231	19	TSP (2)	**K**|IIGG	H	D	S	D	G	G	Trypsin	26,306	9.5	-
74	SerP55 Tequila	QWS65018	1672	245	23	CBM (3), LDL (3), SRCR (2) PAN	**R**|VVRG	H	D	S	D	G	G	Trypsin	26,947	5.9	-

SignalP—Signal peptide; Mm mature—molecular mass of the mature peptidase; pI—isoelectric point of the mature peptidase; TM—transmembrane domain; SerP—serine peptidase. Regulatory domains: Clip—clip domain (IPR022700), classification by [41]; CUB—a domain in Complement 1r/s, Uegf and Bmp1 (IPR000859); Fz—Frizzled domain (IPR020067); LDL—Low-Density Lipoprotein receptor type A repeats (IPR002172); SRCR—Scavenger Receptor Cysteine-Rich domain (IPR017448); TSP—thrombospondine domain (IPR000884); CBM—Chitin-Binding Module (IPR002557); PAN—Plasminogen-Apple-Nematode domain (IPR003609). The amino acid residues after which the propeptide is cleaved are highlighted in bold.

### 2.3. Chymotrypsin-like Peptidases

Thirty insect chymotrypsin-like peptidases are quite diverse in configuration of amino acid residues at positions 189, 216, and 226, which are essential to ensure primary substrate specificity. There was no residues configuration found in the classical vertebrate A-type chymotrypsin P00766 (S189, G216, G226) (Table 2). The bottom of the S1 specificity pocket (sequence position 189) was mostly occupied by G residues, as well as by five classical S, three A, and unique T. In 20 peptidases, where G was present at position 189, S residue was detected in wall positions 216 or 226, and in two sequences (SerP71 and SerP303), A residue was detected like in bovine chymotrypsin B P00767 (S189, G216, A226). Two sequences, SerP16 and SerP69, resembled bovine chymotrypsin-like elastase 2a Q29461 (S189, G216, S226). SerP69 was previously purified and was similar in substrate specificity to chymotrypsins, but did not hydrolyze short substrates containing up to two amino acid residues [27,29], which is typical for insect chymotrypsins [42].

Ten peptidases with a charged residue in the wall of the S1 specificity pocket (GGD, GSD, AGD, GAD) represent another specific to insects group of chymotrypsins, and according to the available biochemical data, display preferential hydrolysis of chymotrypsin substrates [38,43,44]. However, presence of a negatively charged residue at position 226 of the S1 pocket may provide additional specificity for basic side chains at P1 of the substrate due to differences in the overall structure of the S1 pocket, as it was described for crab collagenases brachyurins [45,46].

**Figure 3:**
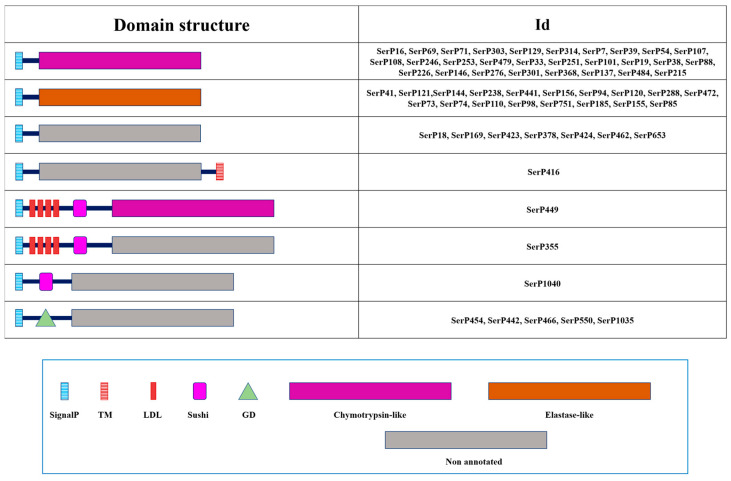
Domain organization of 30 chymotrypsin-like peptidases, 18 elastase-like peptidases, and 15 non-annotated peptidases of *T. molitor*. Regulatory domains are marked with different shapes and colors. Description for domains: SignalP—signal peptide; TM—transmembrane domain; LDL—Low-Density Lipoprotein receptor class A repeat; Sushi—Sushi domain; GD—Gastrulation Defective domain.

Most of the 30 chymotrypsin-like sequences identified in *T. molitor* represented SPs without regulatory domains, except only a single mosaic peptidase (SerP449) with four LDL and one Sushi (IPR000436) domains in propeptide (Figure 3), which was proposed as a putative ortholog of *M. sexta* HP14 (modular SP, MSP) [17]. For most of these chymotrypsin-like SPs, a conserved propeptide cleavage site was predicted (R#I), suggesting trypsins involvement in activation. Alternatively, cleavage at the proposed unique site (H#I) may provide a strictly specific activation (SerP16), or other chymotrypsin- or elastase-like SPs may perform cleavage at the L#I site as in the case of SerP449. Most remarkable was the absence of a canonical activation cleavage site in SerP586, which proposes alternative mechanisms for activation at the L#K site. Most of the chymotrypsin-like SPs had a pI in the acidic region, from 3.8 to 5.3 pH units. Two SPs (SerP101 and SerP276) had a neutral pI and only SerP69 had an alkaline pI of 8.8.

### 2.4. Elastase-like Peptidases

A group of 18 predicted *T. molitor* SP sequences with bulky hydrophobic residues (mostly V or I) at wall position 216 of the S1 binding subsite were annotated as elastase-like enzymes (Table 3). This position is considered a key determinant of the specificity of vertebrate elastases and ensures hydrolysis of small amino acid residues at position P1—A, V, and less commonly, L [47]. The other wall position 226 of the S1 specificity pocket was occupied by the S residue, with the exception of two proteins with residues N (SerP94) and A (SerP472), and at the bottom position 189, there were also small residues G, S, and one A (SerP156). The larger residues were found only at position 216 in three predicted enzymes: T in SerP185, F in SerP155, and Y in SerP85, and the two latter enzymes are of a special interest as its substrate-binding pocket theoretically should be more reduced in depth as compared to other *T. molitor* elastases. Unfortunately, there were no vertebrate peptidases described providing a similar residues configuration of the S1 pocket, to further speculate about their specificity. Elastases with two bulky residues in key positions of the specificity pocket, like bovine pancreatic elastase 1 (A189/V216/T226, Q28153), were absent in *T. molitor*, so it can be assumed that in the majority of insect elastases, the substrate-binding subsite is less occluded compared to that of pancreatic elastases 1 of vertebrates. Another interesting feature of the studied elastases was the presence of I in the position 216 and five SPs had the triad GIS in the S1 subsite, which is typical only for representatives of the Tenebrionidae family. 

All elastase-like enzymes had no regulatory regions in the propeptide (Figure 3), with a conserved propeptide cleavage site (R#I) suggesting for most of the sequences (16 out of 18) involvement of trypsins in activation (Table 3). For only two SPs (SerP94 and SerP120), cleavage at a unique site (H#I) suggests a specific processing pathway. The majority of elastases-like SPs had a pI in the acidic region from 4.0 to 4.9 pH units. A single SP SerP74 had an alkaline pI of 8.6, while vertebrate elastases 1 and 2 are mostly cationic or neutral [48].

### 2.5. Non-Annotated Serine Peptidases

A heterogeneous array of sequences, of which the specificity remains obscure due to the non-typical combination of primary specificity determinant residues, were tentatively grouped as non-annotated SPs, until the biochemical data will become available or closely related orthologs will be found and characterized. A total of 15 sequences were attributed to this group showing the most diverse 189, 216, 226 residues configuration (AAT, GAT, GGK, QGS, RGV, VAD) (Table 4). The propeptide cleavage site in this group of sequences is variable including R, K, L, and I at the C-terminus of the propeptide. Most non-annotated peptidases had neutral or alkaline pI. 

For seven sequences, regulatory regions were identified in the propeptide (Figure 3), including the GD (gastrulation defective, IPR031986) domain confirmed in five related peptidases, which are putative orthologs of *D. melanogaster* gastrulation defective involved in establishment of dorsoventral embryonic polarity [49]. SerP1040 had a Sushi domain, and SerP355 had four LDL and one Sushi. SerP416 had a C-terminal TM domain.

### 2.6. Serine Peptidase Homologs

Serine peptidase homologs are SP-related proteins, for which the functional role is still poorly understood. Although sharing an SP-like domain and fold, they contain one or more substitutions in the catalytic triad residues, suggesting partial or complete loss of catalytic activity, and new functions of SPHs (like regulation, inhibition, and immune modulation) may be compensated through an alternative exosite [50]. In total, 125 SPH sequences with various substitutions of the catalytic triad H57, D102, S195 were identified in *T. molitor* (Table S1). In the catalytic position H57, only 42 proteins had H, and the most common substitution was H195Q in 55 SPHs. At position D102, only 13 substitutions were observed, while S195 was retained in 24 SPHs. In the remaining proteins, S in position 195 was replaced by 26 G, 21 T, 11 N, 10 L, 9 V, 7 I, and also 1–4 residues were presented by A, M, D, E, K, R, Y, F. 

**Figure 4:**
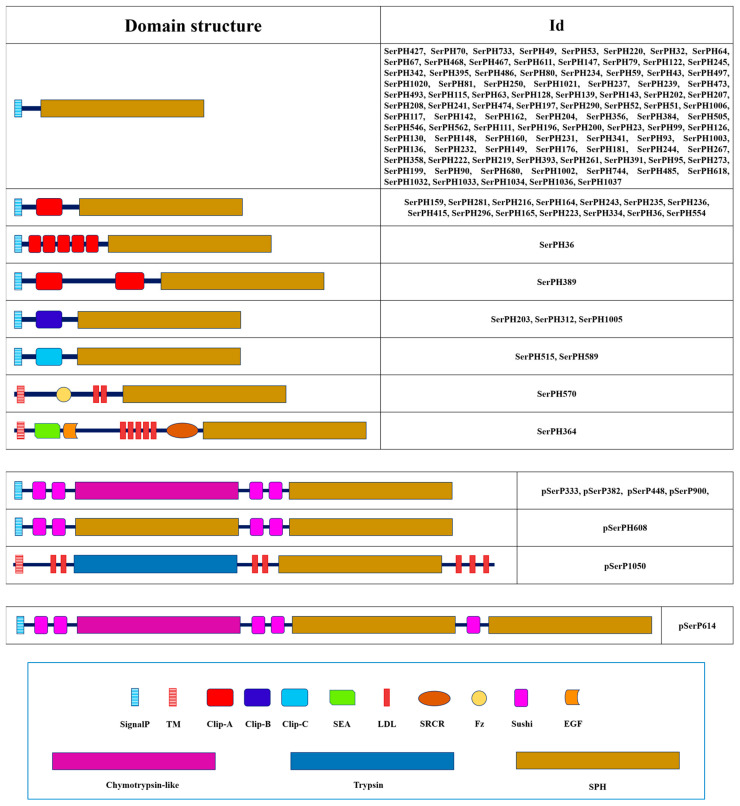
Domain organization of 125 SPHs and 7 polypeptidases of *T. molitor*. Regulatory domains are marked with different shapes and colors. Description for domains: SignalP—signal peptide; TM—transmembrane domain; Clip—Clip domain; SEA—Sperm protein, Enterokinase, and Agrin domain; LDL—Low-Density Lipoprotein receptor class A repeat; SRCR—Scavenger Receptor Cysteine-Rich domain; Fz—Frizzled domain; Sushi—Sushi domain; EGF—laminin/Epidermal Growth Factor-like domain.

Most SPHs had a signal peptide (that is, they are secreted proteins) and are presumably processed by trypsin. In addition, a significant group of proproteins with an unconventional type of processing was also identified, and in some cases, it was even difficult to identify the sequence of the processing site, which is highly conserved in SPs. Most SPHs were anionic proteins with pI at 4–5 pH units. However, a significant proportion of homologs, mainly SPHs with regulatory domains in the propeptide, had neutral or alkaline pI. Most of the SPHs (102 sequences) had no regulatory regions in the propeptide, while 21 out of the rest of the 23 sequences possessed an array or clip domains of A, B, and C types (Figure 4). Two homologs (SerPH570 and SerPH364) were proposed to be associated with plasma membrane via a type-II transmembrane motif. Their prolonged extracellular region included an array of domains such as characteristic juxtamembrane SEA (Sperm protein, Enterokinase, and Agrin domain, IPR000082) or Frizzled domains as well as modules for protein–protein interaction including LDL, EGF (laminin/Epidermal Growth Factor-like domain, IPR002049), and SRCR. 

### 2.7. Polypeptidases

We identified seven *T. molitor* polypeptidase transcripts that encoded putative proteins comprising two to three tandemly arranged peptidase domains, which contained regulatory regions located upstream of each peptidase unit, most often presented by two Sushi domains (Figure 4, Table 5). Four of these proteins contained two peptidase-like domains of which the first (N-terminal) was chymotrypsin-like SP, while the second (C-terminal) was SPH. Another related polypeptidase (pSerPH608) contained two SPH domains, and pSerP614 comprised one chymotrypsin-like and two SPH domains. For all these six secreted proteins was predicted a conserved activation site (L#I) upstream of each SP/SPH domain. And a single transcript encoded a membrane-anchored protein (pSerP1050) containing trypsin and unusual SPH domain with on the whole seven LDL regulatory regions. 

Based on data on “polyserases”, human polypeptidases, it can be assumed that upon activation, peptidase domains may be linked to each other by interdomain disulfide bonds [51]. It was also proposed that SPH domains of secreted polyserases would act as dominant negative binding proteins, modulating the function of the first active SP domain. The same proteolytic mechanism can be proposed for *T. molitor* polypeptidases that resemble human polyserases.

### 2.8. Phylogenetic Analysis of SPs and SPHs in T. molitor

**Figure 5:**
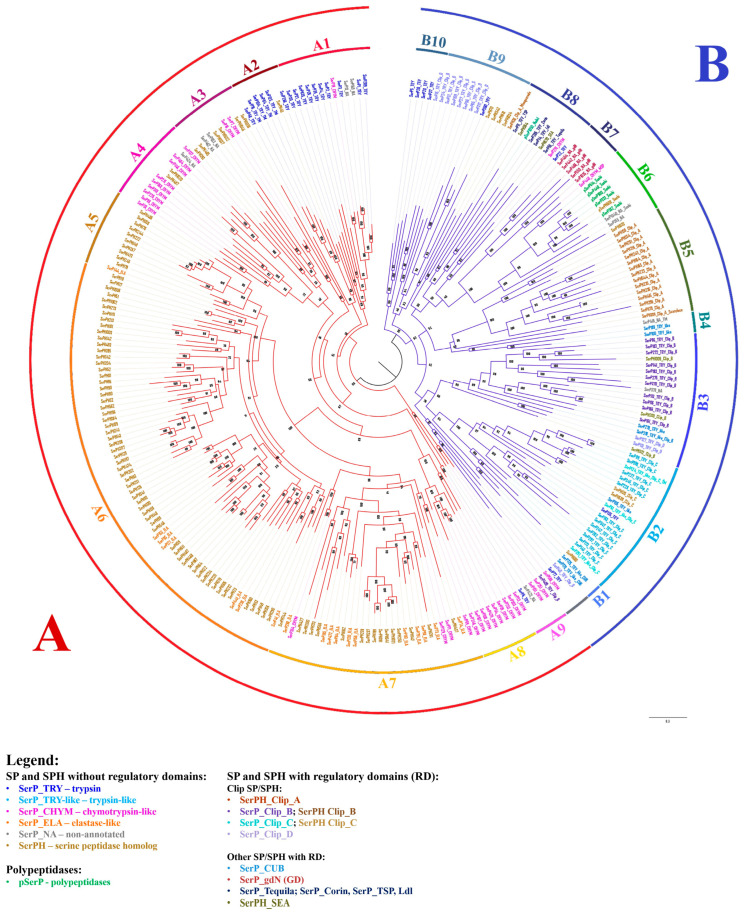
Phylogenetic analysis of 269 SPs and SPHs of *T. molitor*. Complete protein sequences were aligned using MAFFT. The phylogenetic tree was built in the IQTREE service. Peptidases in the tree are divided into two groups: (**A**) (red)—SP and SPH without regulatory domains; (**B**) (blue)—SP and SPH with regulatory domains (including polypeptidases). For the interpretation of the colors of the identifiers, see the legend above.

Phylogenetic analysis of 269 SP-related sequences identified in *T. molitor* showed that they were clustered into two major groups, A and B (Figure 5). Group A (164 sequences) with nine major branches identified (A1–A9) included both SPs and SPHs without regulatory domains in the propeptide. The A1 clade mainly consisted of trypsins including the major digestive trypsin SerP1 (see Section 2.9.3), with only a few sequences proposed as chymotrypsin-like and non-annotated peptidases. Clade A2 included putative trypsins and a single homolog (SerPH43) with a carboxy-terminal hydrophobic extension that resembles a corresponding region of vertebrate peptidases prostasin and testisin, which are post-translationally modified via a glycophosphatydylinositol (GPI) linkage responsible for cell–surface association of these SPs [52,53]. Additionally, two SPs with extended hydrophobic C-terminus from clades A9 (SerP423) and B4 (SerP416) also likely represent distinct GPI-anchored enzymes with unknown specificity. Here, for the first time, we present a group of putative insect analogs of vertebrate regulatory GPI-anchored SPs, of which prostasin also shared a trypsin-like specificity [54]. In some SP sequences, the hydrophobic regions were longer, and they were confidently predicted as TM by programs such as Phobius (SerP125, SerP84, SerP76, SerP416), while in other sequences, predictions about these regions were only from the TM DOCK program (SerP48, SerP104, SerPH43) or had rather low probability. The A3 and A4 clades included predominantly chymotrypsin-like peptidases and related homologs. Chymotrypsin-related sequences from clade A4 represent another insect-specific group containing the acidic residue D226 located on the wall of the S1 pocket (see Section 2.3), but displaying chymotrypsin specificity [38,43,44] in contrast to crab homologs with the same S1 subsite triad [45,46], which efficiently hydrolyze both trypsin and chymotrypsin substrates. It is interesting to note that most of the related homologs from clades A3 and A4 also shared acidic (D or E) residues at position 226 of their primary specificity pocket (Table S1). Clades A5, A6, and A7 included numerous SPHs, likely evolved by multiple duplication events. All 18 predicted elastase-like SPs were scattered among the 87 SPHs, which similar to the elastases mostly shared large aliphatic residues (V/I) at position 216 of their S1 binding pocket. In clade 7, there were also four chymotrypsin-like SPs, one of which, SerP69, was the major digestive chymotrypsin, had an S1 binding subsite (SGS) similar to bovine chymotrypsin-like elastase 2a Q29461, and was biochemically shown to lack the ability to cleave short peptide substrates [27,29] in contrast to another digestive chymotrypsin-like enzyme SerP38 from clade A4 [44]. Clade A8 contained putative chymotrypsin-like SPs mainly with GGS primary specificity determinant. The A9 clade also included chymotrypsin-like SPs, but with the GSG structure of the S1 binding subsite, as well as trypsin SerP6 and unusual non-annotated peptidase SerP423; all these SPs were characterized by acidic pI. 

Group B contained 105 sequences of which most possessed one or more regulatory domains in the extended propeptide. Clip domains represent the most abundant non-catalytic structural units predicted for 60 of such sequences, divided into four major groups (clip-A, -B, -C and -D) based on clip sequence similarity [41]. Fifteen clip-A proteins exclusively represented by non-active SPHs were clustered together into a single clade B5 including prophenoloxidase (pPO)-activating factor II PPAF II (SerPH415) [55]. Clip-A domain folds as irregular β-sheet [56], which is likely characteristic for all of these related SPHs. Clip-B and clip-C proteins from clades B3 and B2, respectively, mainly presented by trypsins and few SPHs, likely shared a more typical clip domain fold composed of antiparallel distorted β-sheet flanked by two α-helices [57]. It is established that clip-C SPs activate terminal clip-B peptidases of the extracellular immune signaling pathway, which cleave the effector molecules pPO or procytokine proSpätzle [41]. In *T. molitor*, these peptidases were identified [58] and clip-C trypsin (SerP228) named Tm-SAE is in clade B2. Clip-C SerP228 activates terminal clip-B trypsin Tm-SPE (SerP183) from clade B3, which in turn activates pPO and its inactive cofactor SerPH415 (clade B5), or proSpätzle in the Toll signaling pathway [4]. It must be noted that one clip-B SP from clade B3 (SerP275) contained two clip-B domains. Clip-D trypsins mainly located in clade B9 possessed a propeptide highly variable in length and sequence (108–548 aa) often including prolonged disordered regions downstream of the N-terminal clip domain. A clip-D peptidase HP1 of *M. sexta* is proposed as an unusual component of immunity associated with the signaling pathway [59]. 

The B1 clade included two trypsin-like peptidases with the CUB domain in propeptide. Shown to be involved in protein–protein interaction, CUB domain(s) are characteristic for an array of chymotrypsin family SPs such as mammalian complement subcomponents (C1r/C1s), enterokinase, and matriptase. Confirmed to be essential for a diverse range of functions from immune regulation to digestion, development, and morphogenesis in vertebrates [60,61], the role of the CUB domain SPs in insects still needs further research. A highly supported clade B6 contained peptidases with Sushi domains including the majority of polypeptidases and chymotrypsin-like modular SP Tm-MSP (SerP448) that initiates proteolytic signaling cascades activating clip-C trypsin Tm-SAE (SerP228) from clade B2 [4,62]. The clade B7 contained five peptidases with the gastrulation defective (GD) domain. In *D. melanogaster* embryo, GD SP participates in the developmental Toll signaling pathway [63]. The clade B8 included sequences of long SP-related proteins with a highly variable set of regulatory domains in the propeptides such as Tequila (SerP55), Corin (SerP285), Nudel (pSerP1050), TSP (SerP11), and membrane-associated homologs SerPH364 and SEA (SerPH570). The clade B10 contained predominantly low-expressed trypsins at the stages of embryogenesis and metamorphosis (see Section 2.9.1 and Section 2.9.2). Interestingly, in a tree constructed using only peptidase domain sequences without prepropeptides (Figure S1), major branches with minor variations are retained, including a clade containing the peptidases with the longest propeptides (B8).

### 2.9. Expression Profiling of SP and SPH Genes in Different Life Stages of T. molitor

**Figure 6:**
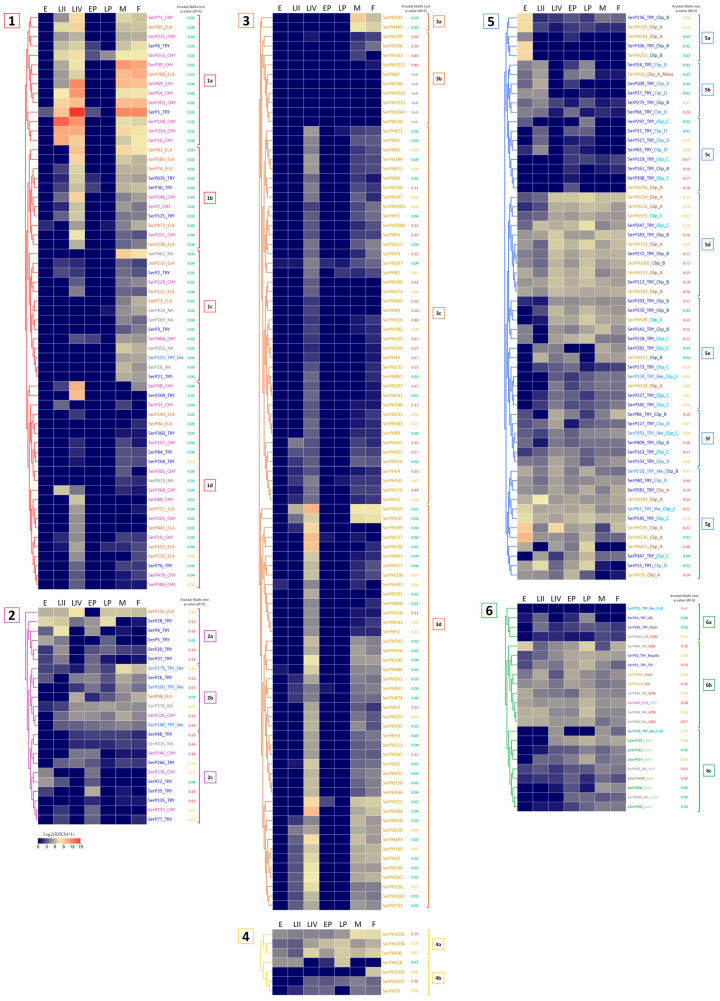
Heatmaps of stage-specific expression pattern of 269 SP/SPH transcripts of *T. molitor*. The hierarchical clustering of RPKM values was used to compare the relative expression levels of transcripts from different *T. molitor* life stages transcriptomes, differentiated into 6 distinct groups. Groups 1–4—SP/SPH without regulatory domains in the propeptide; groups 5–6 have regulatory domains. Group 1 (1a–d) (red)—SPs expressed in feeding stages, group 2 (2a–c) (purple)—SPs expressed at the stages of development, metamorphosis or also at other stages of the life cycle, group 3 (3a–d) (orange)—SPHs expressed at feeding stages; group 4 (4a,b) (yellow)—SPHs expressed at the stages of development, metamorphosis or also at other stages of the life cycle, group 5 (5a–g) (blue)—SPs and SPHs containing clip domains; group 6 (6a–c) (green)—SPs, SPHs, and polypeptidases containing other than clip regulatory domains. The level of mRNA expression is presented as a heatmap from blue to red (log_2_(RPKM + 1)). The resulting *p*-values were adjusted using the Benjamini and Hochberg approach [64]. Values *p* < 0.05 are colored green, indicating the significance of differences in the expression at different stages of *T. molitor* development; values from 0.05 to 0.1 are colored yellow; values greater than 0.1 are colored red, showing the unreliability of differences in the expression values at different stages of *T. molitor* development. The colors of SP/SPH names indicate the types of SPs: trypsins (TRY)—blue, trypsin-like, (TRY-like)—light blue, chymotrypsin-like, (CHYM)—purple, elastase-like, (ELA)—orange, (NA)—non-annotated, grey, pSerp—polypeptidases, TM—transmembrane domain. Designations for regulatory domains: Clip-A—brown; Clip-B—blue; Clip-C—light blue; Clip-D—grey-blue; Sushi—green; GD—red; MSP—blue-green; peptidases with several regulatory domains—dark blue. Life cycle stages: E—egg, LII—second instar larvae, LIV—four instar larvae, EP—early pupa, LP—late pupa, M—male, F—female.

To infer the functional role of the described diversity of SPs/SPHs in various physiological processes, we analyzed expression patterns of their transcripts at different stages of the *T. molitor* life cycle, including eggs, larvae of the II instar, larvae of the IV instar, early and late pupae, and male/female adults. Data for the most highly expressed transcripts at the egg, pupal and feeding larval and adult stages are presented in Table 6, Table 7 and Table 8, respectively, while the expression data for all transcripts are shown as heatmaps in Figure 6, where they are combined into six groups. Group 1—SPs without regulatory domains, expressed at the feeding stages of larvae and adults; group 2—SPs without regulatory domains, expressed in eggs and pupae; group 3—SPHs without regulatory domains, expressed at the feeding stages; group 4—SPHs without regulatory domains, expressed in eggs and pupae; group 5—SPs/SPHs with clip domains; group 6—SPs/SPHs with other regulatory domains.

#### 2.9.1. Embryonic Stage: Eggs

Most of the SPs/SPHs with relatively high mRNA expression levels in the embryonic stage belonged to regulatory proteins, as they contained regulatory clip and GD domains (Table 6, Figure 6(5a,g,6b)). The maximum level of expression in eggs was observed for clip-A SPHs, SerPH236, and Ser PH235, with lower levels at other stages. Transcripts with egg-specific expression showed slightly lower expression levels. Those included clip-B trypsins (SerP166 and SerP116) and SPH (SerPH203), as well as a clip-A SPH SerPH165. Clip-C SPs with moderate expression (SerP145 and SerP61) as well as rather low-expressed SPs with a GD domain (SerP550 and SerP442) demonstrated constitutive expression across most of the stages with the predominance in eggs.

Transcripts without identified regulatory domains in the propeptide with rather low expression levels (Table 6, Figure 6(2a,c)), as well as two SPs with GD domains (SerP466 and SerP454) and clip-A SerPH389, also demonstrated constitutive expression including the egg stage, but with increased levels in the late pupae and IV instar larvae. It should be noted that within this group, three trypsins (SerP28, SerP22, and SerP5) had extended propeptides, but without known regulatory regions, which may indicate the possible presence of potential regulatory domains that have not yet been identified, and, accordingly, specific functions that have not yet been defined. And the only SP in this group with a short propeptide without regulatory domains was a putative elastase SerP156, which could be involved in hydrolytic functions in the egg, such as vitellin hydrolysis.

#### 2.9.2. Metamorphosis: Early and Late Pupae

Most of the highly expressed SP/SPH transcripts at the pupal stages, as well as at the egg stage, contained regulatory domains, and among them, the majority were SPHs with a clip-A domain (Table 7, Figure 6(5d)). In general, SP/SPH transcripts were expressed at both pupal stages, but the levels of expression were higher at the late pupae, and most of the transcripts were also expressed at varying levels across the entire life cycle. The exception was the transcript of the anionic trypsin SerP35 (Table 7, Figure 6(2a)) with a short propeptide, which was specific only for the early pupal stage, and clip-A SerPH78 (Table 7, Figure 6(5g)), which was expressed predominantly at the early pupae. But the highest level of expression at the early pupae was observed for the transcript of homologs SerPH164 with a clip-A domain and SerPH1034 (Table 7, Figure 6(4a)) without regulatory domains, which were upregulated at the late pupae. Noticeable levels of expression were observed here also for transcripts of the SerPH364 homolog and the SerP55 Tequila peptidase (Table 7, Figure 6(6b)), both with a large number of regulatory domains in the propeptide. 

At the late pupae in contrast to the early pupae, trypsin SerP28 (Table 7, Figure 6(2a)) had the highest level of expression together with two peptidases with regulatory domains, SerP247 (Table 7, Figure 6(5d)) and SerP466 (Table 7, Figure 6(6b)). The latter belonged to unannotated peptidases, had a GD regulatory domain, and was also actively expressed at the egg stage. The only transcript that was actively expressed at the late pupal stage and was not expressed in the early pupae belonged to the single elastase-like SerP156 (Table 7, Figure 6(2a)) without a regulatory domain. This type of non-regulated peptidases, SerP156 and SerP35 specific for the early pupae, may be involved in specific tissue remodeling at specific pupal stages. Interestingly, SerP156, as well as trypsin SerP28, were among the highly expressed peptidases at the egg stage, and their transcripts were also upregulated at larval stages IV and II, respectively. 

#### 2.9.3. Feeding Stages: Larvae and Imago (Adults)

The largest part of the SP/SPH transcripts was expressed at the feeding stages, larvae (II and IV instars) and adults (females and males) (Table 8, Figure 6(1,3)), whereas at the developmental stages, eggs and pupae, their genes were practically silent, which most likely indicates the involvement of these SPs/SPHs in the digestive process. This involvement is also confirmed by the data on the high level of expression of these transcripts in the larval gut transcriptome (Table 8). Almost all these transcripts coded for preproenzymes with a small propeptide without regulatory regions. In most cases, they were processed by trypsin after C-terminal R of the propeptide. The highest levels of expression were from active SPs (Table 8, Figure 6(1)), although highly expressed transcripts at feeding stages were also present in the large group of SPHs (Table 8, Figure 6(3)).

Among 61 transcripts of SPs with the classical catalytic triad HDS expressed at one or more feeding stages (Figure 6(1)), several subgroups could be distinguished with similar expression profiles. Subgroup 1a—SPs with a high level of transcripts expression at all feeding stages; 1b—SPs with a high level of expression at IV instar larvae and imago stages; 1c—SPs expressed only at adult stages; 1d—SPs with a high level of transcripts expression mainly at the IV instar larvae.

Subgroup 1a contained the most highly expressed transcripts of digestive SPs (Figure 6(1a)). The majority of them (10) encoded chymotrypsin-like SPs including the earlier characterized major digestive chymotrypsin SerP69 with an extended binding site [29], two transcripts encoded trypsins including the major digestive trypsin SerP1 [28], and two were elastase-encoding transcripts (SerP85, SerP288). The transcript of chymotrypsin-like SerP108 was characterized by an extremely high level of expression at the early larval stage (Table 8). A similar expression profile was demonstrated by chymotrypsin-like SerP314 and trypsin SerP16. All SPs from subgroup 1a had a pI in the acidic region, with the exception of the major trypsin SerP1 and chymotrypsin SerP69 (Section 2.2 and Section 2.3).

Transcripts from SPs of subgroup 1b expressed at IV instar larvae and adults (Figure 6(1b)) encoded five putative elastase-like SPs, three chymotrypsin-like and three trypsins. The most highly expressed were two elastase-like peptidases, SerP41 and SerP185, and chymotrypsin-like SerP246. The majority of SPs from subgroup 1b also had pI in the acidic region, with the exception of elastase-like SerP74 and trypsin SerP30 (Section 2.2 and Section 2.4). Another trypsin, SerP125, had a C-terminal TM domain.

Transcripts from subgroup 1c encoded SPs expressed predominantly at adult stages. Almost half of the group (five) were non-annotated SPs due to an atypical set of amino acid residues in the S1 subsite (Figure 6(1c), Table 4). The subgroup also included two chymotrypsin-like SPs, three elastase-like, three trypsins, and one trypsin-like SP. All these transcripts had a moderate level of expression with maximum values in non-annotated SerP462 (S1 binding subsite TSF). Interestingly, all non-annotated SPs had alkaline or neutral pI (Section 2.5), while all the other SPs were anionic. 

Most of transcripts from subgroup 1d coded for SPs expressed predominantly at the IV instar larvae (Figure 6(1d)). The subgroup included 10 chymotrypsin-like, 6 elastase-like SPs, 5 trypsins, and one non-annotated SP. The maximum level was observed for chymotrypsin-like SerP38 with an unusual S1 binding subsite GGD, but exhibiting substrate specificity typical of chymotrypsins (Table 8) [44]. Another transcript with a high level of expression encoded trypsin SerP209. The remaining transcripts had a moderate or low level of expression. All SPs including the non-annotated one had a PI in the acidic region. Three trypsins (SerP76, SerP84, SerP104) with low levels of transcript expression had a C-terminal TM domain (Section 2.2). 

It must be noted that we found two peptidases with regulatory domains expressed only at the feeding stages: trypsin SerP282 with clip-C domain and trypsin-like SerP178 with a CUB domain (Figure 6(5e,6c)).

Thus, group 1 of 61 SPs (Figure 6(1)) was related to digestion since their transcripts were expressed predominantly at feeding stages, and included the majority of identified chymotrypsin-, elastase-like, and non-annotated SPs without regulatory domains. At the same time, only about a half of non-regulatory trypsins have a similar connection with digestion. The general trend of digestive SPs expression level increase from early to the late larvae instars previously documented [65,66] was confirmed here regarding the expression of transcripts encoding the major digestive SPs of *T. molitor* larvae. Only a few SP transcripts were predominantly expressed at the early larval stage including three chymotrypsin-like enzymes: major SerP108, SerP314, and SerP16 (Table 8, Figure 6(1a)).

In addition to transcripts of active SPs with the classical catalytic active center, 95 transcripts coding for SPHs were predominantly expressed at feeding stages (Table 8, Figure 6(3)) and most of them can be associated with digestive function. The majority of these SPHs, as well as SPs expressed at feeding stages, had a small propeptide without regulatory domains, being processed to mature form by trypsin. The majority of the SPH transcripts were significantly upregulated at the IV larval instar, and the most highly expressed are summarized in Table 8. Almost all SPH transcripts were also confirmed in adults although with lower levels, and only about a quarter of the transcripts was also expressed at the II instar larvae. Two SPH transcripts (SerPH393 and SerPH485) had a significant level of expression only at the adult stage (Figure 6(3a)), while no transcripts specific to the II instar larvae were identified. Note that among the highly expressed SPHs (Table 8), there are SerPH122 and SerPH245 with conservative Ser/Thr substitution in the active center in contrast to the radical replacements in the other SPHs. Characterization of recombinant SerPH122 showed that this synonymous homolog had low but reliably detectable proteolytic activity towards chymotrypsin and trypsin chromogenic peptide substrates [33]. 

The exact role of SPHs is still poorly understood. Nevertheless, whole genome microarray analysis of *T. castaneum* larvae revealed that the transcripts of ten SPH genes were upregulated more than 5-fold as compensation for the effects of cysteine and serine peptidases dietary inhibitors [67]. Also, according to the Section 2.8. mention of the role of clip-A SerPH415 (PPAF-II) in activation of pPO [55], it may be speculated that the above-described major SPHs induced in the feeding stages are somehow involved in luminal digestive SPs activation. 

#### 2.9.4. Constitutively Expressed SP-Related Proteins of *T. molitor*

Another important group of transcripts included SPs/SPHs expressed at several or all stages of the beetle life cycle and presumably participated in important physiological processes such as immune defense, adhesion, regulation of development, and metabolism. Most of the SPs/SPHs with a sufficiently high level of expression at all or most of the life cycle stages had regulatory regions in the sequence structure (Figure 6(5d–g,6b)), and only about one third of the transcripts lacked regulatory domains (Figure 6(2,4)), the majority of which were active SPs. 

SPs/SPHs expressed at all stages included the ones with a clip domain of different subtypes (clip-A, clip-B, clip-C, and clip-D) (Figure 6(5d–g)). The majority of peptidases with the Sushi domain were also expressed at all or most stages of the life cycle. They included polypeptidases and MSP-like SPs (chymotrypsin-like SerP449 and non-annotated SerP355) containing a Sushi domain and four LDL domains (Figure 6(6b,c)). Peptidases containing the GD domain had similar expression profiles. Four out of five of them (SerP442, SerP454, SerP466, SerP550) were expressed at all stages of development with their higher level at the egg and pupa stages, and SerP466 was also found to be highly expressed in adult females. Trypsins, which had a complex multidomain structure, were also expressed at most stages of the life cycle. These peptidases included SerP55 (Tequila), SerP285 (Corin), and SerP11 (TSP). 

## 3. Discussion

SP-related proteins of S1A family identified in the *T. molitor* transcriptome include 269 sequences of which 137 were identified as active SPs with classical catalytic residues, and 125 were annotated as putative non-active SPHs that possess one or more substitutions in the catalytic triad. Seven deduced sequences containing several SP/SPH domains were putative polypeptidases, for which the physiological role remains generally unknown. *T*. *molitor* SPs/SPHs of the S1A chymotrypsin family occupy an intermediate position among insects in terms of the number of identified sequences. A comparable number of SP-related sequences (257) was described for *D. melanogaster* (Diptera: Brachycera) [12], whereas in mosquitoes *A. aegypti* and *A. gambiae* (Diptera: Nematocera), genome-wide analysis identified 369 and 337 SP-related sequences, respectively [12,15]. A significantly lower number with only 44 identified sequences of putative SPs/SPHs was described in *A. mellifera* (Hymenoptera) [23].

In *T. molitor*, 84 SPs and 102 SPHs without regulatory domains constitute the largest group of SP-related proteins. Transcripts of 61 SPs were expressed only in the feeding life stages; 24 of them were highly expressed in the larval gut and presumably play an important role in digestion. Similar quantitative data were previously obtained for other insects including larvae of *D. melanogaster* (53 gut peptidases of which 35 were highly expressed) [12], *A. gambiae* (63 and 27, respectively) [14], and *M. sexta* (61 and 35, respectively) [18]. But even closely related insects have functional differences in the general set of digestive SPs; for example, the most highly expressed SP in *T. molitor* is trypsin SerP1, and in *T. castaneum* it is chymotrypsin XP_970603.1, although their major digestive cysteine peptidases are orthologs with 74% identity [68]. At the same time, there is a close link between the primary structure of the certain digestive SPs and their functions. Accordingly, a comparison of two orthologous pairs of *T. molitor* and *T. castaneum* chymotrypsin-like digestive SPs, SerP38 and CBC01177 (pair I, respectively), and SerP88 and CBC01166 (pair II, respectively), shows that pair I was expressed at the larval and adult stages, while pair II was expressed only in the larval gut [69].

The remaining 23 transcripts of SPs without regulatory domains showed constitutive or specific expression at certain stages of *T. molitor* development. The physiological role of most of these SPs requires further study, but it can be assumed that SPs showing high expression at the egg stage participate in the hydrolysis of storage proteins, as was previously shown for *B. mori* [1], while the SPs expressed at the pupal stages of *T. molitor* can be involved in the breakdown of the larval structures during metamorphosis.

In addition to SPs, the largest group of 95 *T. molitor* SPH sequences lacking regulatory regions were also expressed predominantly during feeding stages. The physiological role of SPHs is still poorly understood; however, some of them highly expressed (9 out of 95) during the feeding stages may play a certain regulatory role that may be related with digestive peptidase activation or their interaction with substrates or inhibitors in the midgut lumen. It was shown that some of the homologs are able to bind with the substrates and even provide a low-rate hydrolysis [33,50].

Another group of SP-related proteins identified included 53 sequences of SPs and 23 SPHs with regulatory domains, such as different clips, LDL, SRCR, TSP, and others. While having significantly lower expression levels than that of the gut digestive peptidases, most of them demonstrated constitutive expression throughout the entire life cycle, while specific SPs and SPHs with various regulatory domains demonstrated increased expression at eggs or pupae stages.

Among these sequences, clip SPs/SPHs were the most numerous. Of the 60 SPs/SPHs with a clip domain that we identified in *T. molitor*, 16 belonged to the clip-A type (all SPHs), 16 to clip-B (13 SPs and 3 SPHs), 17 to clip-C (15 SPs and 2 SPHs), and 11 to clip-D (all SPs). A total number of 60 clip SPs/SPHs is close to 54 sequences identified in the closely related *T. castaneum* [12,34], and about twice the amount of clip SPs/SPHs, including a distinct subtype clip-E SPs, was identified in mosquitoes *A. aegypti* and *A. gambiae* [3,14]. According to the available data, SPs/SPHs with clip domains are non-digestive and are present in the hemolymph of insects and other arthropods. They play an important role in regulation of various physiological processes in insects like innate immune responses leading to activation of pPO necessary for melanization, activation of the Toll-dependent signaling pathway leading to synthesis of antimicrobial peptides [41] or regulation of the dorsal–ventral pattern in *D. melanogaster* embryos [70], as well as regulate the coagulation cascade during hemolymph clotting in crabs [71].

The majority of *T. molitor* clip-containing transcripts were expressed at all or most stages of the ontogeny, but three of them were specific to the egg stage (SerP116 and SerP166 and SerPH203), while at the pupae stage, only increased expression of constitutively expressed clip transcripts was observed. The egg stage specificity of SerP116 and SerP166 was also described by Wu et al. [34] using RT-PCR analysis. The only experimental data on the specific roles of clip SPs/SPHs in *T. molitor* came from B.L. Lee’s laboratory, where the extracellular larval activation cascade of the Toll receptor and pPO was characterized in detail [4,55,58,62]. The proteolytic part of the cascade starts with SerP449 with multiple regulatory domains (MSP), which activates the downstream proSerP228 with clip-C domain (proSAE), which in turn activates proSerP183 (proSPE) with clip-B involved in proSpätzle or pPO activation, but processing of pPO requires additional activation of clip-A homolog proSerPH415.

The remaining smaller part of *T. molitor* SPs/SPHs had different regulatory domains. Transcripts of SPs with a GD domain were expressed constitutively throughout the entire *T. molitor* life cycle including eggs and pupae, and all of them were from the non-annotated group of SPs. Similar peptidases with the GD domain were well studied in *D. melanogaster*, but for the egg stage only [49,63,70]. The stable constitutive mRNA expression of these peptidases in *T. molitor* transcriptomes indicates their possible participation in a wide range of physiological processes in addition to the expected involvement in the cascades forming embryonic polarity during egg development. Another transcript of a large SP Tequila (SerP55) with a variety of regulatory domains was upregulated during the *T. molitor* pupal and adult stages, and in *D. melanogaster* this SP was found throughout development participating in immunity response [72].

One of the most interesting groups in *T. molitor* were polypeptidases, mainly expressed at the pupal and adult stages. Six of them comprise two or three SP/SPH domains and several Sushi domains (Sushi(2)-SP(H)-Sushi(2)-SPH(-Sushi-SPH)). A similar domain architecture, including several peptidase domains and several Sushi domains, has a peptidase SP14 in *T. castaneum* [12]. In *A. gambiae*, several polypeptidases with a little different structure were identified (SP(H)-SPH-clipE-SPH) [14]. In addition, a polypeptidase Nudel (pSerP1050) was also found in *T. molitor*, which contained two peptidase domains—trypsin and an SPH domain with LDL domains. Similar Nudel (LDL(2)-SP-LDL(2)-SPH-LDL(3)) peptidases were identified in many insects [12,14,21]. In *D. melanogaster* embryo, Nudel initiates the peptidase cascade related with dorsal–ventral patterning [70]. Thus, complex polypeptidases were found in insects, but this issue requires further study in order to accurately identify the structure and functions of such proteins. 

The great diversity and abundance of serine peptidases of the chymotrypsin S1A family in various insects provide great opportunities for a more detailed study of insects important for agriculture and/or medicine, and for a fundamental understanding of their physiology. We hope that our study will allow scientists to move in this direction.

## 4. Materials and Methods

### 4.1. Preparation of Biological Material, RNA Isolation and cDNA Sequencing

Whole-body transcriptomes from different stages of the life cycle of *T. molitor* were obtained from the laboratory colony at the Lomonosov Moscow State University (Moscow, Russia), maintained on milled oat flakes at 26 ± 0.5 °C and 75% relative humidity, 0L:24D. Insects were subcultured from the stock colony to obtain specific life stages. Larvae of the II and IV instars were collected one and five weeks post hatch. Not yet pigmented early pupae were sampled immediately after the moult and at the half of the pupal instar (at 10 days post moult). Adults used for the analysis were two weeks after eclosion (males and females separately). Eggs were sifted out of diet 24–48 h after oviposition. Eggs, larvae II and larvae IV, early and late pupae, adult males were collected in two independent biological samples, and adult females were taken in three replicates. RNA was extracted using the RNEasy Mini kit (Qiagen, Hilden, Germany). Immediately prior to isolation, the samples were homogenized by trituration in liquid nitrogen. The concentration of isolated RNA was measured on a Qubit (Thermofisher, Waltham, MA, USA) fluorimeter using a set of reagents for high-sensitivity RNA analysis. The integrity of the RNA was checked by capillary electrophoresis on a Bioanalyzer 2100 (Agilent, Santa Clara, CA, USA). The NEBNext RNA Library Prep Kit for Illumina (New England Biolabs, Ipswich, MA, USA) was used to prepare the libraries according to the recommended protocol with a fragmentation time of 5 min. Sequencing of *T. molitor* developmental stages libraries was performed on an Illumina HiSeq 2000 (Lomonosov Moscow State University, Moscow, Russia) using the TruSeq SBS Kit v3 reagent kit (200 cycles) with the following settings: read length 101, index read length 7, reverse reading length 101. The preprocessed samples contained from 7 million to 24 million reads.

Preparation of biological material, RNA isolation, and cDNA sequencing for gut transcriptome data from *T. molitor* larvae were performed as described earlier [68]. Approximately 240 million sequence reads were obtained, with an approximate 250 bp insert. 

### 4.2. Transcriptomes Assembly

Three different types of *T. molitor* transcriptome assemblies were used in the research.

#### 4.2.1. Assembly of Larval Gut Sequences

Assembly of *T. molitor* larval gut sequences was performed de novo with SeqManNGen (v. 4.0.1.4, DNAStar, Madison, WI, USA) as described in [68]. It included NCGR assembly from all replicates, resulting in 197,800 contigs (N50 = 2232) combined with previous databases of Sanger sequencing [27] and pyrosequencing [30] of mRNA from the larval gut.

#### 4.2.2. Assemblies of Different Developmental Stages

In the transcriptomes of different developmental stages, the quality of the reads was assessed by the MultiQC program (https://multiqc.info) (accessed on 17 November 2023) [73] and preprocessed in Trimmomatic to remove adapters and filter short and low-quality reads (ILLUMINACLIP:TruSeq3-SE:2:30:10, MINLEN:30, SLIDINGWINDOW:5:20) [74]. The reads were mapped to the total transcriptome of *T. molitor* using HISAT2 [75] with mapped reads rate ranging from 84% to 93%. Assembly of transcripts was performed by the Cufflinks program [76] and abundance estimation was assessed with StringTie (-B option) [77].

#### 4.2.3. The Total *T. molitor* Transcriptome Assembly

The total *T. molitor* transcriptome assembly was performed with SeqManNGen (v 15.0.0.160, default parameters) and included the gut assembly (240 million reads) (Section 4.2.1) combined with the Illumina sequencing data obtained for *T. molitor* developmental stages (Section 4.2.1) (628 million reads). There were 342,592,161 total reads assembled, with 143,807,206 reads not assembled and 382,435,025 removed during sampling due to read depth. Reads were assembled into 130,559 contigs, with 36,463 contigs of the length greater than 1 kb.

### 4.3. SP/SPH Identification in the Transcriptomes

BLAST [78] was used to identify ORFs homologous to those encoding SP/SPH. The sequence of human trypsin 2 (UniProt AC P07478) was used as a query and further identified *T. molitor* SP/SPH from different groups were used as queries to search for new sequences. Multiple sequence alignment with BioEdit (v. 7.0.5) [79] was used to refine and build consensus sequences, and in the case of SNPs, the amino acid chosen was the highest percentage and more than 50% of the total. ORFs that were grouped into blocks with identity of at least 95% and that overlapped with another block of at least 10 amino acid residues were considered as referring to a unique peptidase. The resulting sequences were compared with those available in three newly sequenced *T. molitor* genome versions (PRJNA820846: GCA_027725215.1; PRJNA579236: GCA_014282415.3; PRJEB44755: GCA_907166875.3) [80,81].

### 4.4. Analysis of Protein Sequences

Positions of propeptide cleavage site, active site, and S1 substrate-binding subsite residues were predicted by sequence homology through alignment with mature human trypsin 2 (UniProt AC P07478) using BioEdit and Clustal Omega multiple sequence alignment tool (https://www.ebi.ac.uk/Tools/msa/clustalo/) (accessed on 1 April 2024) [82]. Signal peptide was predicted with SignalP 5.0 server (https://services.healthtech.dtu.dk/services/TMHMM-2.0) (accessed on 1 April 2024) (https://services.healthtech.dtu.dk/services/SignalP-5.0/) (accessed on 1 April 2024) [83]. Transmembrane region was predicted with TMHMM Server (v.2.0) (https://services.healthtech.dtu.dk/services/TMHMM-2.0/) (accessed on 1 April 2024) [84], Phobius webserver [85], and TMDOCK server (https://membranome.org/tmdock) (accessed on 5 April 2024) [86]. Domain structure was analyzed using InterProScan (http://www.ebi.ac.uk/interpro/) (accessed on 1 April 2024) [87] and NCBI CDD databases (http://www.ncbi.nlm.nih.gov/Structure/cdd/docs/cdd_search.html) (accessed on 1 April 2024) [88]. Clip domains were identified in the InterProScan; however, some clip domains were identified manually by checking the amino acid sequence of the protein for the presence of Cys doublet in the region close to the peptidase or peptidase-like domain and with four additional Cys residues upstream of the doublet. This combination was designated as clip [41]. The molecular mass and isoelectric point of the mature enzyme of the predicted protein was computed using ExPASy server (https://web.expasy.org/compute_pi/) (accessed on 1 April 2024) [89]. To annotate the substrate specificity of SPs, the sequences were aligned and divided into several types (trypsins, trypsin-like, chymotrypsin-like, elastase-like, and non-annotated) according to the residues in S1 substrate-binding subsite at positions 190, 216, and 226 (chymotrypsin numbering) [9].

### 4.5. Phylogenetic Analysis

Multiple SP/SPH sequence alignments were performed using the MAFFT version 7 (https://mafft.cbrc.jp/alignment/server/) (accessed on 5 April 2024) [90] with default parameters. The phylogenetic tree was constructed using maximum likelihood method by IQ-TREE server in ultrafast mode with 1000 repetitions [91]. FigTree 1.4.4 (http://tree.bio.ed.ac.uk/software/figtree/) (accessed on 5 April 2024) was used to visualize the phylogenetic trees.

### 4.6. Expression Profiling of SP and SPH at Different Developmental Stages

The expression values were calculated for assembled and refined sequences of complete peptidase mRNAs obtained from *T. molitor* transcriptomes and genomes (Section 4.3). To obtain expression values for peptidase mRNA by normalized reads per kilobase per million mapped reads (RPKM) [92], a custom script was used using tBLASTn, calculating each multiread as one unit. RPKM values in biological repeats were averaged for each stage of the life cycle. The transcript of eukaryotic translation factor 3 subunit B (NCBI ID: CAH1377306) was used as a housekeeping protein. Hierarchically clustered gradient heat maps of log2(RPKM+1) values were plotted using TBtools [93]. A Kruskal–Wallis test [94] was conducted among the life stages (*df* = 5), calculated from total RPKM values on Statistics Kingdom webserver (https://www.statskingdom.com/index.html) (accessed on 25 March 2024) [95]. The resulting *p*-values were adjusted using the Benjamini and Hochberg approach [64].

## 5. Conclusions

Serine peptidases (SPs) and homologs (SPHs) of the S1A family constitute a very diverse family of mostly secreted proteins involved in a variety of processes including digestion as well as development and innate immunity regulation. A thorough analysis of several transcriptomes and two newly sequenced genomes of *T. molitor* allowed us to update available information and identify 269 SPs and SPHs in this insect, performing sequence analysis and annotation, constructing phylogenetic relationships, and evaluating expression patterns across the entire life cycle. For 122 SPs, their putative trypsin-, chymotrypsin- and elastase-like specificities were predicted from the S1 binding subsite sequence analysis, and for 15 non-annotated SPs, specificity remains obscure, due to peculiarities of their S1 subsite structure. All studied SP-related sequences of *T. molitor* were grouped according to the organization of their propeptide region. The largest group of 84 SPs and 102 SPHs had no regulatory domains, while the remaining 53 SPs and 23 SPHs had different regulatory domains in the propeptide. Transcripts of 61 SPs without regulatory domains were expressed only in the feeding life stages likely being involved in digestion. The remaining 23 transcripts of SPs without regulatory domains showed mostly constitutive expression while those upregulated at the egg and pupa stages may be involved in the hydrolysis of storage proteins and in the breakdown of the larval structures during metamorphosis, respectively. In addition to SPs, the largest group of 95 *T. molitor* SPH sequences lacking regulatory regions were also expressed predominantly during feeding stages and their physiological role is presumably related to the digestive process; in particular, it may be an interaction with substrates or inhibitors in the midgut lumen. 

The group of SPs and SPHs with regulatory domains contained in the propeptide four types of clips (A–D), GD, Sushi, LDL, SEA, PAN, FZ, TSP, EGF, CUB, SRCR, and CBM domains. Transcripts from the majority of these proteins were expressed constitutively throughout the entire life cycle of *T. molitor*, while some of them were specific to the egg stage or/and upregulated at the pupal stage. For most of these regulatory SP/SPH transcripts, a significantly lower expression level was documented than for the above-described transcripts associated with digestive functions. One of the most interesting groups in *T. molitor* were seven polypeptidases, mainly expressed at the pupal and adult stages. Most of them comprise two or three SP/SPH domains and several Sushi domains. Similar complex polypeptidases were identified in a few insect species, but this group of proteins requires further study in order to accurately identify their structure and functions. The data obtained provide valuable information for further studies on biological functions in insects of the diverse S1A peptidase family.

## Figures and Tables

**Table 2:** Domain organization and key structure features of 30 chymotrypsin-like SPs of *T. molitor*.

№	Name	NCBI ID (Protein)	Preproenzyme/Mature Enzyme (aa)		SignalP (aa)	Regulatory Domain	Propeptide Cleavage Site	Active Site			S1 Subsite			Enzyme Specificity	Mm Mature, Da	pI
1	SerP16	CAH1383061	275	246	16	-	**H**|ITNG	H	D	S	S	G	S	Chymotrypsin-like	25,749	3.9
2	SerP69	ABC88746	275	230	16	-	**R**|IISG	H	D	S	S	G	S	Chymotrypsin-like	22,899	8.8
3	SerP71	CAG9035017	271	235	21	-	**R**|IING	H	D	S	S	G	A	Chymotrypsin-like	24,308	4.1
4	SerP303	CAG9018553	281	237	18	-	**R**|ITGG	H	D	S	S	G	A	Chymotrypsin-like	25,047	4.2
5	SerP129	CAH1365737	265	230	18	-	**R**|IISG	H	D	S	G	A	S	Chymotrypsin-like	24,439	4.0
6	SerP314	ABC88747	266	232	16	-	**R**|IVGG	H	D	S	G	A	S	Chymotrypsin-like	24,475	4.2
7	SerP7	CAG9037665	279	246	16	-	**R**|IING	H	D	S	G	G	S	Chymotrypsin-like	25,707	3.9
8	SerP39	CAG9029806	267	225	16	-	**R**|IIGG	H	D	S	G	G	S	Chymotrypsin-like	23,838	4.3
9	SerP54	CAH1375188	276	233	16	-	**R**|IIGG	H	D	S	G	G	S	Chymotrypsin-like	24,736	4.0
10	SerP107	WJL97990	277	234	16	-	**R**|IIGG	H	D	S	G	G	S	Chymotrypsin-like	25,428	4.3
11	SerP108	CAH1375189	276	233	16	-	**R**|IIGG	H	D	S	G	G	S	Chymotrypsin-like	24,987	3.8
12	SerP246	CAH1375190	275	233	16	-	**R**|IIGG	H	D	S	G	G	S	Chymotrypsin-like	24,822	3.9
13	SerP253	CAH1367742	277	241	21	-	**R**|IIGG	H	D	S	G	G	S	Chymotrypsin-like	25,998	4.1
14	SerP479	WJL97991	276	233	16	-	**R**|IIGG	H	D	S	G	G	S	Chymotrypsin-like	25,012	4.2
15	SerP33	WJL97992	256	217	24	-	**R**|IVGG	H	D	S	G	S	G	Chymotrypsin-like	22,618	4.2
16	SerP251	CAH1372320	255	232	17	-	**R**|IIVG	H	D	S	G	S	G	Chymotrypsin-like	24,576	5.2
17	SerP101	ABC88734	258	235	17	-	**R**|IVNG	H	D	S	G	S	G	Chymotrypsin-like	25,014	6.6
18	SerP19	WJL97993	252	227	16	-	**R**|IVGG	H	D	S	S	S	G	Chymotrypsin-like	23,900	4.5
19	SerP38	QRE01764	258	229	16	-	**R**|VVGG	H	D	S	G	G	D	Chymotrypsin-like	24,410	5.3
20	SerP88	ABC88737	258	229	18	-	**R**|VVGG	H	D	S	G	G	D	Chymotrypsin-like	24,896	5.3
21	SerP226	WJL97994	258	221	22	-	**R**|LIGG	H	D	S	G	G	D	Chymotrypsin-like	23,606	4.2
22	SerP146	CAH1383003	262	222	18	-	**R**|IVGG	H	D	S	G	G	D	Chymotrypsin-like	23,993	4.5
23	SerP276	KAJ3628034	284	247	15	-	**R**|IIHG	H	D	S	G	G	D	Chymotrypsin-like	27,432	6.9
24	SerP301	CAH1380401	244	221	17	-	**R**|IFGG	H	D	S	G	S	D	Chymotrypsin-like	23,620	4.1
25	SerP368	WJL97995	247	233	-	-	**R**|IFGG	H	D	S	A	G	D	Chymotrypsin-like	24,560	4.2
26	SerP137	CAH1379909	248	218	19	-	**K**|IVGG	H	D	S	A	G	D	Chymotrypsin-like	23,683	5.4
27	SerP484	CAH1368908	247	226	16	-	**R**|IVGG	H	D	S	A	G	D	Chymotrypsin-like	24,734	5.0
28	SerP215	CAH1380399	254	231	17	-	**R**|IFGG	H	D	S	G	A	D	Chymotrypsin-like	24,822	4.4
29	SerP586	KAJ3636193	270	224	-	-	**L**|KDNG	H	D	S	T	G	S	Chymotrypsin-like	24,961	5.0
30	SerP449 MSP	BAG14264	632	258	23	LDL (4), Sushi	**L**|IVNG	H	D	S	S	S	G	Chymotrypsin-like	28,757	6.4

SignalP—Signal peptide; Mm mature—molecular mass of the mature protein; pI—isoelectric point of the mature protein; SerP—serine peptidase. Regulatory domains: LDL—Low-Density Lipoprotein receptor (IPR002172); Sushi—Sushi-domain (IPR000436). The amino acid residues after which the propeptide is cleaved are highlighted in bold.

**Table 3:** Domain organization and key structure features of 18 elastase-like SPs of *T. molitor*.

№	Name	NCBI ID (Protein)	Preproenzyme/Mature Enzyme (aa)		SignalP (aa)	Regulatory Domain	Propeptide Cleavage Site	Active Site			S1 Subsite			Enzyme Specificity	Mm Mature, Da	pI
1	SerP41	ABC88760	266	233	19	-	**R**|IVGG	H	D	S	G	I	S	Elastase-like	25,006	4.4
2	SerP121	CAH1368236	274	236	16	-	**R**|IIGG	H	D	S	G	I	S	Elastase-like	26,285	4.5
3	SerP144	WJL97996	268	234	19	-	**R**|IIGG	H	D	S	G	I	S	Elastase-like	25,448	4.4
4	SerP238	KAJ3632560	264	234	21	-	**R**|IVGG	H	D	S	G	I	S	Elastase-like	25,330	4.2
5	SerP441	KAJ3632561	267	234	22	-	**R**|IIGG	H	D	S	G	I	S	Elastase-like	25,072	4.3
6	SerP156	CAH1380384	267	236	19	-	**R**|IING	H	D	S	A	V	S	Elastase-like	25,326	4.6
7	SerP94	WJL97997	266	232	21	-	**H**|IVAG	H	D	S	G	V	N	Elastase-like	24,874	4.8
8	SerP120	WJL97998	268	232	19	-	**H**|IILG	H	D	S	G	V	S	Elastase-like	24,988	4.7
9	SerP288	CAH1375483	266	232	16	-	**R**|IVGG	H	D	S	G	V	S	Elastase-like	24,259	4.0
10	SerP472	CAH1380701	272	235	17	-	**R**|IVNG	H	D	S	S	V	A	Elastase-like	25,265	4.4
11	SerP73	KAJ3638657	267	232	16	-	**R**|IING	H	D	S	S	V	S	Elastase-like	24,485	4.1
12	SerP74	KAH0820461	261	229	16	-	**R**|IING	H	D	S	S	V	S	Elastase-like	23,423	8.6
13	SerP110	KAH0813654	266	231	16	-	**R**|IING	H	D	S	S	V	S	Elastase-like	24,831	4.2
14	SerP98	CAH1365740	267	232	16	-	**R**|IING	H	D	S	S	V	S	Elastase-like	24,969	4.2
15	SerP751	CAH1365741	267	232	16	-	**R**|IING	H	D	S	S	V	S	Elastase-like	24,360	4.0
16	SerP185	KAJ3620429	266	233	17	-	**R**|IING	H	D	S	S	T	S	Elastase-like	24,779	4.9
17	SerP155	KAJ3632649	265	235	16	-	**R**|IIGG	H	D	S	G	F	S	Elastase-like	24,905	4.4
18	SerP85	ABC88761	267	237	16	-	**R**|IIGG	H	D	S	G	Y	S	Elastase-like	25,364	4.3

SignalP—Signal peptide; Mm mature—molecular mass of the mature protein; pI—isoelectric point of the mature protein; SerP—serine peptidase. The amino acid residues after which the propeptide is cleaved are highlighted in bold.

**Table 4:** Domain organization and key structure features of 15 non-annotated SPs of *T. molitor*.

№	Name	NCBI ID (Protein)	Preproenzyme/Mature Enzyme (aa)		SignalP (aa)	Regulatory Domain	Propeptide Cleavage Site	Active Site			S1 Subsite			Enzyme Specificity	Mm Mature, Da	pI	TM (Position)
1	SerP18	WJL97999	258	228	16	-	**K**|IVWG	H	D	S	A	A	T	NA	24,375	8.4	-
2	SerP169	CAH1378761	257	226	16	-	**K**|IVGG	H	D	S	G	A	T	NA	24,300	9.9	-
3	SerP423	CAH1372319	279	257	17	-	**R**|IVNG	H	D	S	G	G	K	NA	28,256	4.5	-
4	SerP416	KAH0820967	300	-	23	-	-	H	D	S	Q	G	S	NA	-	-	277–299
5	SerP378	WJL98000	357	253	22	-	**K**|ISGG	H	D	S	R	G	I	NA	28,551	8.1	-
6	SerP424	KAJ3633461	250	227	18	-	**R**|IIGG	H	D	S	R	G	V	NA	25,210	7.0	-
7	SerP462	KAH0817404	257	-	23	-	-	H	D	S	T	S	F	NA	-	-	-
8	SerP653	CAH1380361	252	-	16	-	-	H	D	S	V	A	D	NA	-	-	-
9	SerP355	WJL98001	551	267	19	LDL (4), Sushi	**L**|IVNG	H	D	S	G	S	T	NA	29,980	5.1	-
10	SerP1040	WJL98002	432	263	22	Sushi	**L**|IING	H	D	S	S	S	S	NA	27,132	7.7	-
11	SerP454	CAH1384889	476	257	15	GD	**L**|ITHG	H	D	S	S	S	V	NA	28,642	7.8	-
12	SerP442	CAH1384890	561	257	17	GD	**L**|ISYG	H	D	S	T	G	I	NA	28,750	7.7	-
13	SerP466	KAJ3628554	427	247	23	GD	**K**|PANE	H	D	S	S	G	V	NA	27,618	7.3	-
14	SerP550	CAH1380129	447	249	18	GD	**L**|VLKG	H	D	S	G	A	I	NA	27,949	8.9	-
15	SerP1035	CAH1380127	568	249	25	GD	**L**|VVNG	H	D	S	G	S	V	NA	27,582	9.7	-

SignalP—Signal peptide; Mm mature—molecular mass of the mature protein; pI—isoelectric point of the mature protein; TM—transmembrane domain; SerP—serine peptidase. Regulatory domains: LDL—Low-Density Lipoprotein receptor (IPR002172); Sushi—Sushi-domain (IPR000436), GD—Gastrulation Defective domain (IPR031986). The amino acid residues after which the propeptide is cleaved are highlighted in bold.

**Table 5:** Domain organization and key structure features of seven polypeptidases of *T. molitor*.

№	Name	NCBI ID (Protein)	Preproenzyme (aa)	SignalP (aa)	Regulatory Domain	Propeptide Cleavage Site	Active Site			S1 Subsite			Enzyme Specificity
1	pSerP448	WKK29891	892	20	Sushi (2)	**L**|IVGG	H	D	S	S	S	G	Chymotrypsin-like
					Sushi (2)	**L**|IVKG	H	D	A	S	S	A	SPH
2	pSerP900	CAH1380589	891	22	Sushi (2)	**L**|IVGG	H	D	S	S	S	G	Chymotrypsin-like
					Sushi (2)	**L**|IVKG	H	D	A	S	S	A	SPH
3	pSerP333	CAH1382424	891	24	Sushi (2)	**L**|IVSG	H	D	S	S	S	G	Chymotrypsin-like
					Sushi (2)	**L**|IVNG	R	N	V	F	Q	V	SPH
4	pSerP382	WKK29892	837	23	Sushi (2)	**L**|IVGG	H	D	S	S	A	G	Chymotrypsin-like
					Sushi (2)	**L**|IIGG	Q	D	R	I	S	G	SPH
5	pSerPH608	WKK29893	895	23	Sushi (2)	**L**|IVGG	H	D	G	S	S	G	SPH
					Sushi (2)	**L**|IIGG	Y	D	G	S	F	T	SPH
6	pSerP614	WKK29894	1347	24	Sushi (2)	**L**|IVNG	H	D	S	S	S	A	Chymotrypsin-like
					Sushi (2)	**L**|IING	H	D	G	S	S	S	SPH
					Sushi	**L**|IVNG	Q	D	S	A	S	A	SPH
7	pSerP1050 Nudel	CAH1374346	1830	TM (58–80)	LDL (7)	**R**|VVGG	H	D	S	D	G	G	Trypsin
						**N**|ITSQ	T	E	D	D	S	A	SPH

SignalP—Signal peptide; pSerP(SerPH)—serine (serine peptidase homolog) polypeptidase. Regulatory domains: LDL—Low-Density Lipoprotein receptor (IPR002172); Sushi—Sushi domain (IPR000436). Replacements in the active center are marked in grey. The amino acid residues after which the propeptide is cleaved are highlighted in bold.

**Table 6:** *T. molitor* SP/SPH transcripts with the highest expression levels at the egg stage compared to other stages.

									Expression, RPKM						
Sequence Name	Regulatory Domains	Active Site			S1 Subsite			Annotation of Sequence	Eggs	Larvae II	Larvae IV	Early Pupae	Late Pupae	Males	Females
SerPH236	Clip_A	H	D	G	D	G	G	SPH	**1001**	27	26	16	57	8	9
SerPH235	Clip_A	H	D	G	D	G	G	SPH	**518**	16	360	33	44	4	71
SerPH203	Clip_B	H	D	G	D	G	A	SPH	**357**	0	0	0	0	0	0
SerP166	Clip_B	H	D	S	D	G	G	Trypsin	**344**	0	0	0	0	0	0
SerPH165	Clip_A	H	D	G	D	G	G	SPH	**331**	4	1	3	4	2	2
SerP116	Clip_B	H	D	S	D	G	G	Trypsin	**329**	2	3	6	3	13	9
SerP145	Clip_C	H	D	S	D	G	G	Trypsin	**150**	53	37	61	89	53	57
SerP28	-	H	D	S	D	G	G	Trypsin	**147**	114	13	32	186	0	1
SerP466	GD	H	D	S	S	G	V	N/A	**122**	10	26	37	139	6	62
SerP61	Clip_C	H	D	S	D	G	A	Trypsin-like	**119**	41	44	41	60	23	32
SerP156	-	H	D	S	A	V	S	Elastase-like	**65**	68	321	0	106	113	91
SerP454	GD	H	D	S	S	S	V	N/A	**61**	65	25	22	93	24	23
SerP550	GD	H	D	S	G	A	I	N/A	**61**	56	32	34	54	13	25
SerP442	GD	H	D	S	T	G	I	N/A	**53**	19	16	12	45	24	21
SerPH389 Scarface	Clip_A_	H	D	Y	D	D	G	SPH	**51**	200	29	42	27	27	43
SerP5	-	H	D	S	D	G	G	Trypsin	**47**	17	0	0	9	0	0
SerP22	-	H	D	S	D	G	G	Trypsin	**34**	6	0	7	0	0	0

Bold indicates RPKM values for the egg stage.

**Table 7:** *T. molitor* SP/SPH transcripts with the highest expression levels at the early and late pupal stages compared to other stages.

									Expression, RPKM						
Sequence Name	Regulatory Domains	Active Site			S1 Subsite			Annotation of Sequence	Eggs	Larvae II	Larvae IV	Early Pupae	Late Pupae	Males	Females
SerPH164	Clip_A	H	D	G	D	G	G	SPH	6	10	85	**88**	123	72	78
SerPH1034	-	Q	N	T	E	E	K	SPH	9	7	35	**70**	92	31	53
SerP247	Clip_C	H	D	S	D	G	G	Trypsin	9	73	48	**65**	161	19	34
SerP145	Clip_C	H	D	S	D	G	G	Trypsin	150	53	37	**61**	89	53	57
SerPH159	Clip_A	H	D	G	D	G	A	SPH	29	9	105	**60**	121	37	70
SerPH78	Clip_A	H	D	G	D	G	G	SPH	27	24	11	**58**	7	0	1
SerP35	-	H	D	S	D	G	G	Trypsin	4	0	0	**58**	0	1	0
SerP228	Clip_C	H	D	S	D	G	G	Trypsin	17	0	35	**52**	80	15	14
SerPH364	SEA; EGF; LDL; SRCR	S	D	E	D	R	R	SPH	15	52	7	**52**	27	16	29
SerPH243	Clip_A	H	D	G	D	G	A	SPH	42	22	67	**51**	43	44	51
SerP55 Tequila	CBM (3), LDL (3), SRCR (2) PAN	H	D	S	D	G	G	Trypsin	5	10	25	**51**	58	46	40
SerP113	Clip_B	H	D	S	D	G	G	Trypsin	27	35	82	**49**	75	45	34
SerPH223	Clip_A	H	D	G	D	G	G	SPH	44	15	73	**46**	86	19	33
SerPH216	Clip_A	H	D	G	D	G	G	SPH	2	5	50	**46**	73	64	31
SerP11 TSP	TSP (2)	H	D	S	D	G	G	Trypsin	5	23	14	**44**	72	25	10
SerP28	-	H	D	S	D	G	G	Trypsin	147	114	13	32	**186**	0	1
SerP247	Clip_C	H	D	S	D	G	G	Trypsin	9	73	48	65	**161**	19	34
SerP466	GD	H	D	S	S	G	V	N/A	122	10	26	37	**139**	6	62
SerPH164	Clip_A	H	D	G	D	G	G	SPH	6	10	85	88	**123**	72	78
SerPH159	Clip_A	H	D	G	D	G	A	SPH	29	9	105	60	**121**	37	70
SerPH415	Clip_A	H	D	G	D	G	G	SPH	47	0	34	14	**118**	0	0
SerP15	Clip_D	H	D	S	D	G	G	Trypsin	35	142	9	35	**113**	1	0
SerP156	-	H	D	S	A	V	S	Elastase-like	65	68	321	0	**106**	113	91
SerPH680	-	H	N	I	S	G	T	SPH	17	7	115	30	**98**	61	105
SerPH589	Clip_C	R	D	S	D	G	A	SPH	0	1	47	41	**94**	43	39
SerP454	GD	H	D	S	S	S	V	N/A	61	65	25	22	**93**	24	23
SerPH1034	-	Q	N	T	E	E	K	SPH	9	7	35	70	**92**	31	53
SerP145	Clip_C	H	D	S	D	G	G	Trypsin	150	53	37	61	**89**	53	57
SerPH223	Clip_A	H	D	G	D	G	G	SPH	44	15	73	46	**86**	19	33
SerPH618	-	S	D	G	V	Q	G	SPH	26	27	0	2	**85**	0	0

Bold indicates RPKM values for the pupae stages.

**Table 8:** *T. molitor* SP/SPH transcripts with the highest expression levels at the feeding stages compared to other stages and IV instar larvae gut.

								Expression, RPKM							
Name	Active Site			Annotation of Sequence	S1 Subsite			Eggs	Larvae II	Larvae IV	Early Pupae	Late Pupae	Males	Females	Larval IV Gut
SerP1	H	D	S	Trypsin	D	G	G	7	675	**16,880**	6	0	2563	2646	**10,480**
SerP69	H	D	S	Chymotrypsin-like	S	G	S	0	37	**2574**	0	0	782	695	**4107**
SerP108	H	D	S	Chymotrypsin-like	G	G	S	0	6151	**2092**	0	0	191	152	**6195**
SerP54	H	D	S	Chymotrypsin-like	G	G	S	0	255	**1934**	0	0	207	299	**2102**
SerP314	H	D	S	Chymotrypsin-like	G	A	S	0	1207	**1087**	0	0	413	312	**5564**
SerP38	H	D	S	Chymotrypsin-like	G	G	D	0	4	**976**	0	0	0	26	**1517**
SerP209	H	D	S	Trypsin	D	G	G	0	0	**765**	0	0	0	0	**494**
SerP303	H	D	S	Chymotrypsin-like	S	G	A	0	450	**736**	0	0	718	770	**419**
SerP41	H	D	S	Elastase-like	G	I	S	0	0	**678**	0	0	403	294	**8062**
SerP16	H	D	S	Chymotrypsin-like	S	G	S	0	1193	**512**	0	0	114	270	**151**
SerP246	H	D	S	Chymotrypsin-like	G	G	S	0	11	**481**	0	0	20	41	**511**
SerP85	H	D	S	Elastase-like	G	Y	S	0	158	**465**	0	0	93	99	**1394**
SerP185	H	D	S	Elastase-like	S	T	S	0	0	**425**	0	0	117	72	**2417**
SerP71	H	D	S	Chymotrypsin-like	S	G	A	0	30	**378**	0	0	147	100	**991**
SerP253	H	D	S	Chymotrypsin-like	G	G	S	0	160	**331**	6	27	256	271	**517**
SerP156	H	D	S	Elastase-like	A	V	S	65	68	**321**	0	106	113	91	**220**
SerP39	H	D	S	Chymotrypsin-like	G	G	S	0	52	**220**	0	0	1877	1324	**718**
SerP251	H	D	S	Chymotrypsin-like	G	S	G	0	0	**217**	0	0	10	6	**1164**
SerP74	H	D	S	Elastase-like	S	V	S	0	4	**146**	0	0	64	68	**615**
SerP288	H	D	S	Elastase-like	G	V	S	0	37	**140**	0	0	788	1101	**1681**
SerPH219	S	D	V	SPH	G	I	S	0	64	**1243**	0	0	282	295	**3687**
SerPH384	Q	D	S	SPH	G	I	S	0	0	**985**	0	0	52	98	**1813**
SerPH237	Q	D	G	SPH	S	I	S	0	0	**965**	1	0	1	0	**355**
SerPH239	Q	D	G	SPH	S	I	S	0	0	**800**	0	0	0	2	**733**
SerPH122	H	D	T	SPH	G	L	S	0	0	**411**	5	0	103	125	**1223**
SerPH493	Q	D	I	SPH	G	V	S	0	9	**347**	0	0	89	35	**991**
SerPH562	Q	D	S	SPH	G	I	S	0	8	**264**	0	0	51	67	**576**
SerPH245	H	D	T	SPH	G	M	T	0	0	**225**	0	0	37	41	**510**
SerPH290	Q	D	M	SPH	G	R	S	0	6	**207**	0	0	41	35	**106**
SerPH136	Q	D	T	SPH	G	L	S	0	7	**185**	0	0	17	18	**490**

Bold font indicates the RPKM values for larvae IV and larval IV gut. Shaded are replaced amino acids in the catalytic triad of the SPH.

## Data Availability

Raw sequencing data can be accessed through the SRA database. SRA site: https://dataview.ncbi.nlm.nih.gov/object/PRJNA1099774?reviewer=erel6q9bo7vvv7n3ii6hl8c0na (accessed on 1 April 2024).

## Supplementary Materials

The following supporting information can be downloaded at: https://www.mdpi.com/article/10.3390/ijms25115743/s1.

## References

[1] Ikeda M., Yaginuma T., Kobayashi M., Yamashita O. (1991). cDNA cloning, sequencing and temporal expression of the protease responsible for vitellin degradation in the silkworm, *Bombyx mori*. Comp. Biochem. Physiol. B.

[2] Choo Y.M., Lee K.S., Yoon H.J., Lee S.B., Kim J.H., Sohn H.D., Jin B.R. (2007). A serine protease from the midgut of the bumblebee, *Bombus ignites* (Hymenoptera: Apidae): cDNA cloning, gene structure, expression and enzyme activity. Eur. J. Entomol..

[3] Waterhouse R.M., Kriventseva E.V., Meister S., Xi Z., Alvarez K.S., Bartholomay L.C., Barillas-Mury C., Bian G., Blandin S., Christensen B.M. (2007). Evolutionary dynamics of immune-related genes and pathways in disease-vector mosquitoes. Science.

[4] Kan H., Kim C.H., Kwon H.M., Park J.W., Roh K.B., Lee H., Park B.J., Zhang R., Zhang J., Söderhäll K. (2008). Molecular control of phenoloxidase-induced melanin synthesis in an insect. J. Biol. Chem..

[5] Jiang H., Vilcinskas A., Kanost M.R. (2010). Immunity in lepidopteran insects. Adv. Exp. Med. Biol..

[6] Veillard F., Troxler L., Reichhart J.M. (2016). *Drosophila melanogaster* clip-domain serine proteases: Structure, function and regulation. Biochimie.

[7] Clark K.D. (2020). Insect Hemolymph Immune Complexes. Subcell Biochem..

[8] Contreras E.G., Glavic Á., Brand A.H., Sierralta J.A. (2021). The serine protease homolog, scarface, is sensitive to nutrient availability and modulates the development of the *Drosophila* blood-brain barrier. J. Neurosci..

[9] Perona J.J., Craik C.S. (1995). Structural basis of substrate specificity in the serine proteases. Protein Sci..

[10] Bao Y.Y., Qin X., Yu B., Chen L.B., Wang Z.C., Zhang C.X. (2014). Genomic insights into the serine protease gene family and expression profile analysis in the planthopper, *Nilaparvata lugens*. BMC Genom..

[11] Ross J., Jiang H., Kanost M., Wanga Y. (2003). Serine proteases and their homologs in the *Drosophila melanogaster* genome: An initial analysis of sequence conservation and phylogenetic relationships. Gene.

[12] Cao X., Jiang H. (2018). Building a platform for predicting functions of serine protease-related proteins in *Drosophila melanogaster* and other insects. Insect Biochem. Mol. Biol..

[13] Christophides G.K., Zdobnov E., Barillas-Mury C., Birney E., Blandin S., Blass C., Brey P.T., Collins F.H., Danielli A., Dimopoulos G. (2002). Immunity-related genes and gene families in *Anopheles gambiae*. Science.

[14] Cao X., Gulati M., Jiang H. (2017). Serine protease-related proteins in the malaria mosquito, *Anopheles gambiae*. Insect Biochem. Mol. Biol..

[15] Brackney D.E., Isoe J., Black W.C., Zamora J., Foy B.D., Miesfeld R.L., Olson K.E. (2010). Expression profiling and comparative analyses of seven midgut serine proteases from the yellow fever mosquito, *Aedes aegypti*. J. Insect Physiol..

[16] Soares T.S., Watanabe R.M.O., Lemos F.J.A., Tanaka A.S. (2011). Molecular characterization of genes encoding trypsinlike enzymes from *Aedes aegypti* larvae and identification of digestive enzymes. Gene.

[17] Cao X., He Y., Hu Y., Zhang X., Wang Y., Zou Z., Chen Y., Blissard G.W., Kanost M.R., Jiang H. (2015). Sequence conservation, phylogenetic relationships, and expression profiles of nondigestive serine proteases and serine protease homologs in *Manduca sexta*. Insect Biochem. Mol. Biol..

[18] Miao Z., Cao X., Jiang H. (2020). Digestion-related proteins in the tobacco hornworm, *Manduca sexta*. Insect Biochem. Mol. Biol..

[19] Zhao P., Wang G.H., Dong Z.M., Duan J., Xu P.Z., Cheng T.C., Xiang Z.H., Xia Q.Y. (2010). Genome-wide identification and expression analysis of serine proteases and homologs in the silkworm *Bombyx mori*. BMC Genom..

[20] Liu H., Heng J., Wang L., Tang X., Guo P., Li Y., Xia Q., Zhao P. (2020). Identification, characterization, and expression analysis of clip-domain serine protease genes in the silkworm, *Bombyx mori*. Dev. Comp. Immunol..

[21] Lin H., Xia X., Yu L., Vasseur L., Gurr G.M., Yao F., Yang G., You M. (2015). Genome-wide identification and expression profiling of serine proteases and homologs in the diamondback moth, *Plutella xylostella* (L.). BMC Genom..

[22] Yang L., Xing B.Q., Wang L.K., Yuan L.L., Manzoor M., Li F., Wu S. (2021). Identification of serine protease, serine protease homolog and prophenoloxidase genes in *Spodoptera frugiperda* (Lepidoptera: Noctuidae). J. Asia-Pac. Entomol..

[23] Zou Z., Lopez D.L., Kanost M.R., Evans J.D., Jiang H. (2006). Comparative analysis of serine protease-related genes in the honey bee genome: Possible involvement in embryonic development and innate immunity. Insect Mol. Biol..

[24] Yang L., Lin Z., Fang Q., Wang J., Yan Z., Zou Z., Song Q., Ye G. (2017). The genomic and transcriptomic analyses of serine proteases and their homologs in an endoparasitoid, *Pteromalus puparum*. Dev. Comp. Immunol..

[25] Oppert B., Muszewska A., Steczkiewicz K., Šatović-Vukšić E., Plohl M., Fabrick J.A., Vinokurov K.S., Koloniuk I., Johnston J.S., Smith T.P.L. (2022). The Genome of *Rhyzopertha dominica* (Fab.) (Coleoptera: Bostrichidae): Adaptation for Success. Genes.

[26] Tribolium Sequencing Consortium (2008). The genome of the model beetle and pest *Tribolium castaneum*. Nature.

[27] Prabhakar S., Chen M.-S., Elpidina E.N., Vinokurov K.S., Smith C.M., Marshall J., Oppert B. (2007). Sequence analysis and molecular characterization of larval midgut cDNA transcripts encoding peptidases from the yellow mealworm, *Tenebrio molitor* L.. Insect Mol. Biol..

[28] Tsybina T.A., Dunaevsky Y.E., Belozersky M.A., Zhuzhikov D.P., Oppert B., Elpidina E.N. (2005). Digestive proteinases of yellow mealworm (*Tenebrio molitor*) larvae: Purification and characterization of a trypsin-like proteinase. Biochemistry.

[29] Elpidina E.N., Tsybina T.A., Dunaevsky Y.E., Belozersky M.A., Zhuzhikov D.P., Oppert B. (2005). A chymotrypsin-like proteinase from the midgut of *Tenebrio molitor* larvae. Biochimie.

[30] Oppert B., Dowd S.E., Bouffard P., Li L., Conesa A., Lorenzen M.D., Toutges M., Marshall J., Huestis D.L., Fabrick J. (2012). Transcriptome profiling of the intoxication response of *Tenebrio molitor* larvae to *Bacillus thuringiensis* Cry3Aa protoxin. PLoS ONE.

[31] Zhiganov N.I., Tereshchenkova V.F., Oppert B., Filippova I.Y., Belyaeva N.V., Dunaevsky Y.E., Belozersky M.A., Elpidina E.N. (2021). The dataset of predicted trypsin serine peptidases and their inactive homologs in *Tenebrio molitor* transcriptomes. Data Brief.

[32] Gorbunov A.A., Akentyev F.I., Gubaidullin I.I., Zhiganov N.I., Tereshchenkova V.F., Elpidina E.N., Kozlov D.G. (2021). Biosynthesis and Secretion of Serine Peptidase SerP38 from *Tenebrio molitor* in the Yeast *Komagataella kurtzmanii*. Appl. Biochem. Microbiol..

[33] Tereshchenkova V.F., Zhiganov N.I., Akentyev P.I., Gubaidullin I.I., Kozlov D.G., Belyaeva N.V., Filippova I.Y., Elpidina E.N. (2021). Preparation and properties of the recombinant *Tenebrio molitor* SerPH122—Proteolytically active homolog of serine peptidase. Appl. Biochem. Microbiol..

[34] Wu C.Y., Xiao K.R., Wang L.Z., Wang J., Song Q.S., Stanley D., Wei S.J., Zhu J.Y. (2022). Identification and expression profiling of serine protease-related genes in *Tenebrio molitor*. Arch. Insect Biochem. Physiol..

[35] Errico S., Spagnoletta A., Verardi A., Moliterni S., Dimatteo S., Sangiorgio P. (2022). *Tenebrio molitor* as a source of interesting natural compounds, their recovery processes, biological effects, and safety aspects. Compr. Rev. Food Sci. Food Saf..

[36] Moncada-Pazos A., Cal S., Lopez-Otín C., Rawlings N.D., Salvesen G. (2013). Polyserases. Handbook of Proteolytic Enzymes.

[37] Schechter I., Berger A. (1967). On the size of the active site in proteases. I. Papain. Biochem. Biophys. Res. Commun..

[38] Botos I., Meyer E., Nguyen M., Swanson S.M., Koomen J.M., Russell D.H., Meyer E.F. (2000). The structure of an insect chymotrypsin. J. Mol. Biol..

[39] Rawlings N.D., Barrett A.J., Rawlings N.D., Salvesen G. (2013). Introduction: Serine peptidases and their clans. Handbook of Proteolytic Enzymes.

[40] Baird T.T., Craik C.S., Rawlings N.D., Salvesen G. (2013). Trypsin. Handbook of Proteolytic Enzymes.

[41] Kanost M.R., Jiang H. (2015). Clip-domain serine proteases as immune factors in insect hemolymph. Curr. Opin. Insect Sci..

[42] Lopes A.R., Sato P.M., Terra W.R. (2009). Insect chymotrypsins: Chloromethyl ketone inactivation and substrate specificity relative to possible coevolutional adaptation of insects and plants. Arch. Insect Biochem. Physiol..

[43] Whitworth S.T., Blum M.S., Travis J. (1998). Proteolytic enzymes from larvae of the fire ant, *Solenopsis invicta*. Isolation and characterization of four serine endopeptidases. J. Biol. Chem..

[44] Tereshchenkova V.F., Zhiganov N.I., Gubaeva A.S., Akentyev F.I., Dunaevsky Y.E., Kozlov D.G., Belozersky M.A., Elpidina E.N. (2024). Characteristics of recombinant chymotrypsin-like peptidase from the midgut of *Tenebrio molitor* larvae. Appl. Biochem. Microbiol..

[45] Tsu C.A., Perona J.J., Schellenberger V., Turck C.W., Craik C.S. (1994). The substrate specificity of *Uca pugilator* collagenolytic serine protease 1 correlates with the bovine type I collagen cleavage sites. J. Biol. Chem..

[46] Tsu C.A., Craik C.S. (1996). Substrate recognition by recombinant serine collagenase 1 from *Uca pugilator*. J. Biol. Chem..

[47] Bode W., Meyer E., Powers J.C. (1989). Human leukocyte and porcine pancreatic elastase: X-ray crystal structures, mechanism, substrate specificity, and mechanism-based inhibitors. Biochemistry.

[48] Oliveira E.B., Salgado M.C.O., Rawlings N.D., Salvesen G. (2013). Pancreatic elastases. Handbook of Proteolytic Enzymes.

[49] DeLotto R. (2001). Gastrulation defective, a complement factor C2/B-like protease, interprets a ventral prepattern in *Drosophila*. EMBO Rep..

[50] Reynolds S.L., Fischer K. (2015). Pseudoproteases: Mechanisms and function. Biochem. J..

[51] Cal S., Moncada-Pazos A., Lopez-Otin C. (2007). Expanding the complexity of the human degradome: Polyserases and their tandem serine protease domains. Front. Biosci..

[52] Chen L.M., Skinner M.L., Kauffman S.W., Chao J., Chao L., Thaler C.D., Chai K.X. (2001). Prostasin is a glycosylphosphatidylinositol-anchored active serine protease. J. Biol. Chem..

[53] Scarman A.L., Hooper J.D., Boucaut K.J., Sit M., Webb G.C., Normyle J.F., Antalis T.M. (2001). Organization and chromosomal localization of the murine Testisin gene encoding a serine protease temporally expressed during spermatogenesis. Eur. J. Biochem..

[54] Rickert K.W., Kelley P., Byrne N.J., Diehl R.E., Hall D.L., Montalvo A.M., Reid J.C., Shipman J.M., Thomas B.W., Munshi S.K. (2008). Structure of human prostasin, a target for the regulation of hypertension. J. Biol. Chem..

[55] Lee K.Y., Zhang R., Kim M.S., Park J.W., Park H.Y., Kawabata S., Lee B.L. (2002). A zymogen form of masquerade-like serine proteinase homologue is cleaved during pro-phenoloxidase activation by Ca^2+^ in coleopteran and *Tenebrio molitor* larvae. Eur. J. Biochem..

[56] Piao S., Song Y.-L., Kim J.H., Park S.Y., Park J.W., Lee B.L., Oh B.-H., Ha N.-C. (2005). Crystal structure of a clip-domain serine protease and functional roles of the clip domains. EMBO J..

[57] Huang R., Lu Z., Dai H., Velde D.V., Prakash O., Jiang H. (2007). The solution structure of clip domains from *Manduca sexta* prophenoloxidase activating proteinase-2. Biochemistry.

[58] Kim C.H., Kim S.J., Kan H., Kwon H.M., Roh K.B., Jiang R., Yang Y., Park J.W., Lee H.H., Ha N.C. (2008). A three-step proteolytic cascade mediates the activation of the peptidoglycan-induced toll pathway in an insect. J. Biol. Chem..

[59] He Y., Wang Y., Yang F., Jiang H. (2017). *Manduca sexta* hemolymph protease-1, activated by an unconventional non-proteolytic mechanism, mediates immune responses. Insect Biochem. Mol. Biol..

[60] Bork P., Beckmann G. (1993). The CUB domain: A widespread module in developmentally regulated proteins. J. Mol. Biol..

[61] Blanc G., Font B., Eichenberger D., Moreau C., Ricard-Blum S., Hulmes D.J., Moali C. (2007). Insights into how CUB domains can exert specific functions while sharing a common fold: Conserved and specific features of the CUB1 domain contribute to the molecular basis of procollagen C-proteinase enhancer-1 activity. J. Biol. Chem..

[62] Park J.W., Kim C.H., Kim J.H., Je B.R., Roh K.B., Kim S.J., Lee H.H., Ryu J.H., Lim J.H., Oh B.H. (2007). Clustering of peptidoglycan recognition protein-SA is required for sensing lysine-type peptidoglycan in insects. Proc. Natl. Acad. Sci. USA.

[63] Cho Y.S., Stevens L.M., Sieverman K.J., Nguyen J., Stein D. (2012). A ventrally localized protease in the *Drosophila* egg controls embryo dorsoventral polarity. Curr. Biol..

[64] Benjamini Y., Hochberg Y. (1995). Controlling the false Discovery rate: A practical and powerful approach to multiple testing. J. R. Statist. Soc. B.

[65] Keller M., Sneh B., Strizhov N., Prudovsky E., Regev A., Koncz C., Schell J., Zilberstein A. (1996). Digestion of δ-endotoxin by gut proteases may explain reduced sensitivity of advanced instar larvae of *Spodoptera littoralis* to CryIC. Insect Biochem. Mol. Biol..

[66] Zalunin I.A., Elpidina E.N., Oppert B., Soberón M., Gao Y., Bravo A. (2015). The role of proteolysis in the biological activity of Bt insecticidal crystal proteins. Bt Resistance—Characterization and Strategies for GM Crops Producing Bacillus thuringiensis Toxins.

[67] Oppert B., Elpidina E.N., Toutges M., Mazumdar-Leighton S. (2010). Microarray analysis reveals strategies of *Tribolium castaneum* larvae to compensate for cysteine and serine protease inhibitors. Comp. Biochem. Physiol. D Genom. Proteom..

[68] Martynov A.G., Elpidina E.N., Perkin L., Oppert B. (2015). Functional analysis of C1 family cysteine peptidases in the larval gut of *Tenebrio molitor* and *Tribolium castaneum*. BMC Genom..

[69] Broehan G., Arakane Y., Beeman R.W., Kramer K.J., Muthukrishnan S., Merzendorfer H. (2010). Chymotrypsin-like peptidases from *Tribolium castaneum*: A role in molting revealed by RNA interference. Insect Biochem. Mol. Biol..

[70] LeMosy E.K., Tan Y.Q., Hashimoto C. (2001). Activation of a protease cascade involved in patterning the *Drosophila embryo*. Proc. Natl. Acad. Sci. USA.

[71] Muta T., Hashimoto R., Miyata T., Nishimura H., Toh Y., Iwanaga S. (1990). Proclotting enzyme from horseshoe crab hemocytes. cDNA cloning, disulfide locations, and subcellular localization. J. Biol. Chem..

[72] Munier A.I., Medzhitov R., Janeway C.A., Doucet D., Capovilla M., Lagueux M. (2004). Graal: A *Drosophila* gene coding for several mosaic serine proteases. Insect Biochem. Mol. Biol..

[73] Ewels P., Magnusson M., Lundin S., Käller M. (2016). MultiQC: Summarize analysis results for multiple tools and samples in a single report. Bioinformatics.

[74] Bolger A.M., Lohse M., Usadel B. (2014). Trimmomatic: A flexible trimmer for Illumina sequence data. Bioinformatics.

[75] Pertea M., Kim D., Pertea G.M., Leek J.T., Salzberg S.L. (2016). Transcript-level expression analysis of RNA-seq experiments with HISAT, StringTie and Ballgown. Nat. Protoc..

[76] Trapnell C., Williams B., Pertea G., Mortazavi A., Kwan G., van Baren M.J., Salzberg S.L., Wold B.J., Pachter L. (2010). Transcript assembly and quantification by RNA-Seq reveals unannotated transcripts and isoform switching during cell differentiation. Nat. Biotechnol..

[77] Pertea M., Pertea G.M., Antonescu C.M., Chang T.C., Mendell J.T., Salzberg S.L. (2015). StringTie enables improved reconstruction of a transcriptome from RNA-seq reads. Nat. Biotechnol..

[78] Altschul S.F., Gish W., Miller W., Myers E.W., Lipman D.J. (1990). Basic local alignment search tool. J. Mol. Biol..

[79] Hall T.A. (1999). BioEdit: A user-friendly biological sequence alignment editor and analysis program for Windows 95/98/NT. Nucl. Acids. Symp..

[80] Kaur S., Stinson S.A., di Cenzo G.C. (2023). Whole genome assemblies of *Zophobas morio* and *Tenebrio molitor*. G3.

[81] Eriksson T., Andere A.A., Kelstrup H., Emery V.J., Picard C.J. (2020). The yellow mealworm (*Tenebrio molitor*) genome: A resource for the emerging insects as food and feed industry. J. Insects Food Feed.

[82] McWilliam H., Li W., Uludag M., Squizzato S., Park Y.M., Buso N., Cowley A.P., Lopez R. (2013). Analysis Tool Web Services from the EMBL-EBI. Nucleic Acids Res..

[83] Almagro Armenteros J.J., Tsirigos K.D., Sønderby C.K., Petersen T.N., Winther O., Brunak S., von Heijne G., Nielsen H. (2019). SignalP 5.0 improves signal peptide predictions using deep neural networks. Nat. Biotechnol..

[84] Krogh A., Larsson B., von Heijne G., Sonnhammer E.L. (2001). Predicting transmembrane protein topology with a hidden Markov model: Application to complete genomes. J. Mol. Biol..

[85] Käll L., Krogh A., Sonnhammer E.L. (2007). Advantages of combined transmembrane topology and signal peptide prediction--the Phobius web server. Nucleic Acids Res..

[86] Lomize A.L., Hage J.M., Pogozheva I.D. (2018). Membranome 2.0: Database for proteome-wide profiling of bitopic proteins and their dimers. Bioinformatics.

[87] Paysan-Lafosse T., Blum M., Chuguransky S., Grego T., Pinto B.L., Salazar G.A., Bileschi M.L., Bork P., Bridge A., Colwell L. (2023). InterPro in 2022. Nucleic Acids Res..

[88] Wang J., Chitsaz F., Derbyshire M.K., Gonzales N.R., Gwadz M., Lu S., Marchler G.H., Song J.S., Thanki N., Yamashita R.A. (2023). The conserved domain database in 2023. Nucleic Acids Res..

[89] Gasteiger E., Gattiker A., Hoogland C., Ivanyi I., Appel R.D., Bairoch A. (2003). ExPASy: The proteomics server for in-depth protein knowledge and analysis. Nucleic Acids Res..

[90] Katoh K., Rozewicki J., Yamada K.D. (2019). MAFFT online service: Multiple sequence alignment, interactive sequence choice and visualization. Brief. Bioinform..

[91] Trifinopoulos J., Nguyen L.T., von Haeseler A., Minh B.Q. (2016). W-IQ-TREE: A fast online phylogenetic tool for maximum likelihood analysis. Nucleic Acids Res..

[92] Mortazavi A., Williams B.A., McCue K., Schaeffer L., Wold B. (2008). Mapping and quantifying mammalian transcriptomes by RNA-Seq. Nat. Methods.

[93] Chen C., Chen H., Zhang Y., Thomas H.R., Frank M.H., He Y., Xia R. (2020). TBtools: An Integrative Tool kit Developed for Interactive Analyses of Big Biological Data. Mol. Plant.

[94] Kruskal W.H., Wallis W.A. (1952). Use of ranks in one-criterion variance analysis. J. Am. Stat. Assoc..

[95] Statistics Kingdom. https://www.statskingdom.com/index.html.

