# Supplementary material for: The Set of Serine Peptidases of the Tenebrio molitor Beetle: Transcriptomic Analysis on Different Developmental Stages
Source: Int J Mol Sci. 2024 May 25;25(11):5743. doi: 10.3390/ijms25115743 (PMC11172050; doi:10.3390/ijms25115743)
Supplement: Supplementary file 1 [file ijms-25-05743-s001.zip › ijms-2992336-supplementary.pdf]

Supplementary Materials

1 Supplementary Figures

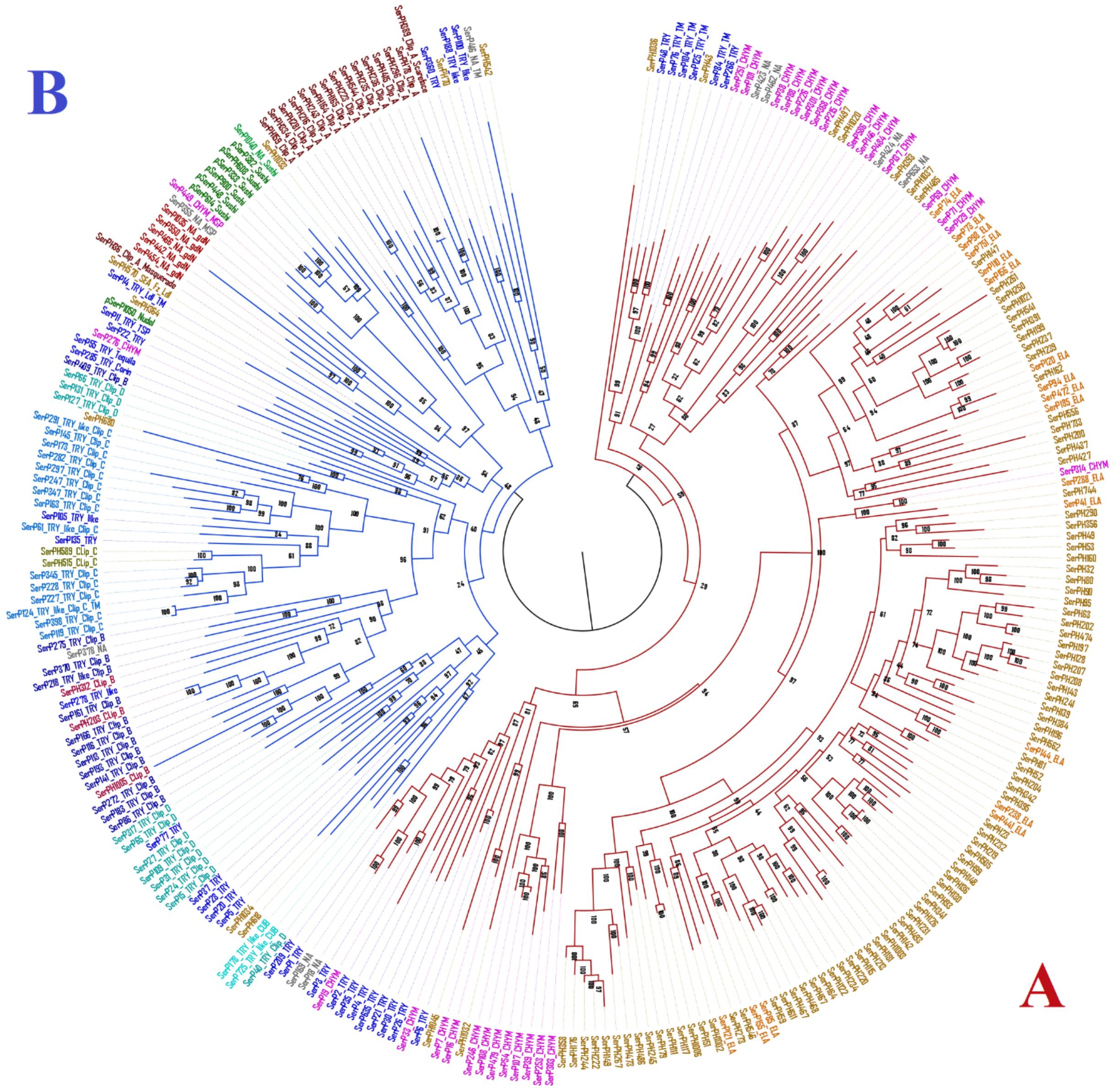

Supplementary Figure S1. Phylogenetic analysis of 269 mature SPs and SPHs sequences of *T. molitor*. Sequences were aligned using MAFFT. The phylogenetic tree was built in the IQTREE service. Peptidases in the tree are divided into two groups: group (A) (red)—SP and SPH without regulatory domains; group (B) (blue)—SP and SPH with regulatory domains (including polypeptidases).

1 Supplementary Tables

Supplementary Table S1. Domain organization and key structure features of 125 SPHs of *T. molitor*.

№	Name	NCBI ID (protein)	Preproenzyme/ Mature Enzyme (aa)		SignalP (aa)	Regulatory domain	Propeptide cleavage site	Active site	S1 subsite	Annotation	Mm mature. Da	pI	TM (position)
1	SerPH427	CAH1378290	264	236	20	-	**Q**|INGG	D N E	G I S	SPH	26,063	8.8	-
2	SerPH70	QRE01765	280	249	19	-	**K**|IVGG	H D D	D G G	SPH	27,206	4.4	-
3	SerPH733	WJL97962	253	228	16	-	**W**|LHYS	H D D	S C M	SPH	25,418	4.7	-
4	SerPH49	CAH1369791	258	234	18	-	**R**|IVGG	H D M	G I S	SPH	25,612	4.4	-
5	SerPH53	CAH1369792	258	234	18	-	**R**|IVGG	H D M	G I S	SPH	25,485	4.3	-
6	SerPH220	KAJ3632626	262	231	23	-	**R**|IIGG	H D N	G I S	SPH	25,169	4.3	-
7	SerPH32	KAJ3632593	261	236	18	-	**R**|IIGG	H D N	G I S	SPH	26,055	4.4	-
8	SerPH64	WJL97963	267	235	23	-	**R**|IIGG	H D N	G I S	SPH	26,241	4.5	-
9	SerPH67	KAJ3632622	263	231	23	-	**R**|IIGG	H D N	G I S	SPH	26,006	4.2	-
10	SerPH468	CAH1368201	268	234	24	-	**R**|IIGG	H D N	G I S	SPH	25,666	4.6	-
11	SerPH467	CAH1368202	268	234	24	-	**R**|IIGG	H D N	G I S	SPH	25,631	4.4	-
12	SerPH611	CAH1368200	265	235	24	-	**R**|IIGG	H D N	G I S	SPH	25,844	4.6	-
13	SerPH147	CAH1365742	269	234	24	-	**R**|IING	H D N	G I S	SPH	25,213	3.8	-
14	SerPH79	CAH1375498	262	235	16	-	**R**|IIGG	H D T	G M T	SPH	25,169	4.7	-
15	SerPH122	CAH1368241	257	231	21	-	**R**|IIGG	H D T	G L S	SPH	25,076	4.2	-
16	SerPH245	CAH1375497	262	233	16	-	**R**|IIGG	H D T	G M T	SPH	24,658	4.6	-
17	SerPH342	KAJ3632571	269	235	17	-	**R**|IIGG	H D T	G I K	SPH	25,492	4.6	-
18	SerPH395	KAJ3632570	269	235	16	-	**R**|IIGG	H D T	G I K	SPH	25,388	4.4	-
19	SerPH486	CAH1375496	263	235	24	-	**R**|IIGG	H D T	G F S	SPH	25,405	4.5	-
20	SerPH80	WJL97964	263	236	17	-	**R**|IIGG	H D T	G I S	SPH	26,156	4.6	-
21	SerPH234	KAJ3632623	263	232	22	-	**R**|IIGG	L D N	G I S	SPH	25,405	4.3	-
22	SerPH59	WJL97965	263	232	21	-	**R**|IIGG	N D N	G I S	SPH	25,291	4.3	-
23	SerPH43	CAH1373500	279	257	16	-	**R**|IAGG	N N N	D G S	SPH	28,285	6.8	-
24	SerPH497	WJL97966	252	224	19	-	**R**|VIGG	Q D A	G A D	SPH	23,826	4.3	-
25	SerPH1020	CAH1380194	248	219	20	-	**R**|IMNG	M D M	A S D	SPH	23,347	3.9	-
26	SerPH81	WJL97967	259	233	17	-	**R**|IIGG	Q D G	G F S	SPH	25,204	4.5	-
27	SerPH250	CAH1365744	262	227	16	-	**R**|IING	Q D G	N I S	SPH	23,917	3.9	-
28	SerPH1021	KAJ3638670	262	227	16	-	**R**|IING	Q D G	N I S	SPH	24,047	3.7	-
29	SerPH237	CAH1365731	265	230	16	-	**R**|ILNG	Q D G	S I S	SPH	23,871	4.2	-
30	SerPH239	ABC88745	265	230	16	-	**R**|ILNG	Q D G	S I T	SPH	23,935	4.1	-
31	SerPH473	KAH0819850	268	237	16	-	**R**|IIGG	Q D I	G I T	SPH	25,672	4.2	-
32	SerPH493	KAJ3632562	269	237	18	-	**R**|IIGG	Q D I	G V S	SPH	25,743	4.3	-
33	SerPH115	KAJ3632565	264	236	13	-	**R**|IIGG	Q D I	G L S	SPH	25,451	4.6	-
34	SerPH63	KAJ3632590	261	232	18	-	**R**|IIGG	Q D L	G I S	SPH	25,023	4.3	-
35	SerPH128	KAH0821415	262	234	17	-	**R**|IVGG	Q D L	G I S	SPH	25,544	4.2	-
36	SerPH139	KAJ3632573	268	235	16	-	**R**|IIGG	Q D L	G I S	SPH	25,199	4.5	-
37	SerPH143	CAH1368222	264	235	18	-	**R**|IIGG	Q D L	G I S	SPH	25,506	4.2	-
38	SerPH202	KAJ3632591	270	241	18	-	**R**|IIGG	Q D L	G I S	SPH	25,711	4.1	-
39	SerPH207	KAJ3632577	262	234	17	-	**R**|IVGG	Q D L	G I S	SPH	25,130	4.4	-
40	SerPH208	CAH1368225	262	234	17	-	**R**|IVGG	Q D L	G I S	SPH	25,492	4.4	-
41	SerPH241	KAJ3632584	264	235	18	-	**R**|IIGG	Q D L	G I S	SPH	25,344	4.2	-
42	SerPH474	WJL97968	280	235	17	-	**R**|IIGG	Q D L	G I S	SPH	25,453	4.1	-
43	SerPH197	KAH0820594	280	235	17	-	**R**|IIGG	Q D L	G I S	SPH	25,346	4.1	-
44	SerPH290	KAH0808032	255	232	16	-	**R**|IVGG	Q D M	G R S	SPH	24,721	4.3	-
45	SerPH52	KAJ3632600	256	233	15	-	**R**|IIGG	Q D S	G F S	SPH	25,262	4.3	-
46	SerPH51	KAJ3632618	267	236	15	-	**R**|IIGG	Q D S	G I S	SPH	25,107	4.5	-
47	SerPH1006	KAJ3632617	262	231	20	-	**R**|IIGG	Q D S	G I S	SPH	24,917	5.2	-
48	SerPH117	KAJ3632616	264	233	25	-	**R**|IIGG	Q D S	G I S	SPH	25,036	5.1	-
49	SerPH142	CAH1368218	263	238	16	-	**R**|IFEG	Q D S	R V S	SPH	26,185	4.3	-
50	SerPH162	CAH1365667	267	232	16	-	**R**|IISG	Q D S	G S Y	SPH	24,745	4.6	-
51	SerPH204	KAJ3632602	261	235	15	-	**R**|IIGG	Q D S	G F S	SPH	25,550	4.5	-
52	SerPH356	KAJ3632559	268	233	17	-	**R**|IIGG	Q D S	G F S	SPH	25,022	4.3	-
53	SerPH384	KAJ3632603	258	237	20	-	**R**|IIGG	Q D S	G I S	SPH	25,694	4.2	-
54	SerPH505	KAJ3632612	269	237	22	-	**R**|IIGG	Q D S	G F S	SPH	25,590	4.5	-
55	SerPH546	WJL97969	263	233	24	-	**R**|IIGG	Q D S	G F S	SPH	24,781	4.2	-
56	SerPH562	CAH1368228	275	235	23	-	**R**|IIGG	Q D S	G I S	SPH	25,309	4.3	-

57	SerPH111	KAJ3632615	257	234	19	-	**R**|IIGG	Q	D	S	G	I	S	SPH	25,491	4.3	-
58	SerPH196	WJL97970	265	238	17	-	**R**|IVGG	Q	D	S	G	I	S	SPH	25,846	4.3	-
59	SerPH200	KAJ3620427	263	227	18	-	**R**|LIDG	Q	D	S	G	W	T	SPH	24,644	4.6	-
60	SerPH23	KAJ3632605	267	236	21	-	**R**|VIGG	Q	D	T	G	I	S	SPH	25,248	4.0	-
61	SerPH99	WJL97971	264	233	20	-	**R**|IIGG	Q	D	T	G	V	S	SPH	25,324	4.5	-
62	SerPH126	KAH0818074	264	233	21	-	**R**|IIGG	Q	D	T	G	I	S	SPH	25,577	4.3	-
63	SerPH130	WJL97972	264	233	20	-	**R**|IIGG	Q	D	T	G	I	S	SPH	25,347	4.6	-
64	SerPH148	KAJ3632607	265	234	19	-	**R**|IIGG	Q	D	T	G	I	S	SPH	25,762	4.3	-
65	SerPH160	CAH1369835	257	234	17	-	**R**|IVGG	Q	D	T	G	L	S	SPH	25,408	4.5	-
66	SerPH231	WJL97973	264	233	21	-	**R**|IIGG	Q	D	T	G	I	S	SPH	25,573	4.4	-
67	SerPH341	WJL97974	264	233	20	-	**R**|IIGG	Q	D	T	G	I	S	SPH	25,291	4.4	-
68	SerPH93	WJL97975	264	233	18	-	**R**|IIGG	Q	D	T	G	I	S	SPH	25,397	4.3	-
69	SerPH1003	CAH1368216	282	248	18	-	**R**|IIGG	Q	D	T	G	V	S	SPH	27,076	4.8	-
70	SerPH136	KAJ3632606	264	234	17	-	**R**|IIGG	Q	D	T	G	L	S	SPH	25,460	4.3	-
71	SerPH232	KAJ3632604	276	237	19	-	**R**|IIGG	Q	D	T	G	V	S	SPH	25,532	4.2	-
72	SerPH149	ABC88750	272	241	16	-	**R**|IVGG	Q	D	V	G	I	T	SPH	26,457	4.3	-
73	SerPH176	KAJ3626463	269	238	16	-	**R**|IVGG	Q	D	V	G	I	T	SPH	26,034	4.3	-
74	SerPH181	WJL97976	259	234	20	-	**R**|IIGG	Q	D	V	G	L	S	SPH	25,585	4.8	-
75	SerPH244	CAH1375491	269	238	16	-	**R**|IVGG	Q	D	V	G	I	T	SPH	25,833	4.3	-
76	SerPH267	WJL97977	270	239	16	-	**R**|IIGG	Q	D	V	G	V	T	SPH	25,973	3.9	-
77	SerPH358	CAH1375486	268	237	16	-	**R**|IVGG	Q	D	V	G	I	T	SPH	26,255	4.3	-
78	SerPH222	ABC88752	269	238	16	-	**R**|IVGG	Q	D	V	G	I	T	SPH	26,137	4.3	-
79	SerPH219	KAJ3632572	261	232	16	-	**R**|IIGG	S	D	V	G	I	S	SPH	24,441	4.2	-
80	SerPH393	WJL97978	249	226	17	-	**R**|ILSD	S	D	S	G	G	D	SPH	24,167	6.3	-
81	SerPH261	CAH1365751	256	223	16	-	**R**|IIGG	S	N	I	G	V	S	SPH	23,751	4.1	-
82	SerPH391	WJL97979	263	227	16	-	**R**|IING	S	N	A	N	I	S	SPH	24,291	4.1	-
83	SerPH95	KAJ3632596	270	237	16	-	**R**|IIGG	T	D	S	G	I	S	SPH	26,106	4.4	-
84	SerPH273	CAH1369790	268	236	19	-	**R**|IIGG	T	D	S	G	V	S	SPH	25,697	4.5	-
85	SerPH199	CAH1365747	265	230	16	-	**R**|IING	V	S	G	T	L	S	SPH	24,763	4.3	-
86	SerPH90	KAJ3632594	268	236	17	-	**R**|IIGG	Y	D	T	G	I	S	SPH	26,009	4.4	-
87	SerPH680	CAH1375940	290	246	20	-	**Q**|WTND	H	N	I	S	G	T	SPH	27,893	9.2	-
88	SerPH1002	KAJ3632569	267	233	16	-	**R**|IIGG	H	N	I	G	L	S	SPH	25,062	4.3	-
89	SerPH744	CAH1375484	264	230	16	-	**R**|IIAG	R	D	S	G	A	S	SPH	24,806	3.9	-
90	SerPH485	WJL97980	296	255	17	-	**R**|MIKG	L	N	Y	G	I	E	SPH	28,321	5.3	-
91	SerPH618	WJL97981	306	248	27	-	**R**|FMGG	S	D	G	V	Q	G	SPH	26,579	4.9	-
92	SerPH1032	CAH1380364	250	-	22	-	-	S	D	R	N	K	E	SPH	-	-	-
93	SerPH1033	WJL97982	267	244	24	-	**R**|CSSN	H	D	E	R	Y	S	SPH	27,263	8.9	-
94	SerPH1034	CAH1373571	312	281	17	-	**K**|CEAG	Q	N	E	E	E	K	SPH	31,315	9.6	-
95	SerPH1036	CAH1376577	345	302	21	-	M|IDGN	T	D	S	Q	N	E	SPH	33,759	4.3	-
96	SerPH1037	KAH0808835	246	-	20	-	-	R	G	S	G	S	T	SPH	-	-	-
97	SerPH542	CAH1377086	280	222	no	-	**S**|LQEH	H	N	Y	K	G	V	SPH	24,685	9.3	7-29
98	SerPH556	KAJ3620428	266	233	18	-	**R**|IING	S	D	S	N	M	A	SPH	25,253	4.1	-
99	SerPH541	CAH1365748	261	226	21	-	**R**|IING	Q	D	G	A	I	S	SPH	24,037	3.7	-
100	SerPH213	KAJ3632609	260	235	20	-	**R**|IIGG	Q	D	V	G	L	S	SPH	25,791	4.8	-
101	SerPH437	CAH1380402	265	239	21	-	**R**|IYGG	G	D	G	A	L	A	SPH	26,030	4.4	-
102	SerPH1045	KAJ3617561	257	235	17	-	**R**|MIGG	H	D	G	A	L	A	SPH	25,649	4.4	-
103	SerPH159	CAH1373087	391	253	22	Clip_A	**R**|ILTP	H	D	G	D	G	A	SPH	28,081	5.6	-
104	SerPH281	CAH1373100	366	246	17	Clip_A	**Q**|SSSY	H	D	G	D	G	S	SPH	27,035	9.7	-
105	SerPH216	QRE01766	348	254	16	Clip_A	**K**|IGND	H	D	G	D	G	G	SPH	28,298	6.1	-
106	SerPH164	KAJ3627136	355	260	20	Clip_A	**R**|IIGD	H	D	G	D	G	G	SPH	28,760	7.7	-
107	SerPH243	KAJ3627138	350	253	18	Clip_A	**I**|GANG	H	D	G	D	G	A	SPH	28,148	7.2	-
108	SerPH235	QRE01767	407	263	16	Clip_A	**K**|ITGN	H	D	G	D	G	G	SPH	28,787	6.0	-
109	SerPH236	KAJ3627133	407	263	16	Clip_A	**K**|ITGN	H	D	G	D	G	G	SPH	28,762	6.1	-
110	SerPH415	BAC15605	444	261	15	Clip_A	**N**|LIGG	H	D	G	D	G	G	SPH	28,676	6.1	-
111	SerPH296	KAJ3627135	387	249	30	Clip_A	**D**|KGAN	H	D	G	G	S	H	SPH	27,879	6.3	-
112	SerPH165	CAH1371357	387	260	21	Clip_A	**K**|IKNP	H	D	G	D	G	G	SPH	29,080	5.8	-
113	SerPH223	CAH1373089	400	264	21	Clip_A	**R**|ITGN	H	D	G	D	G	G	SPH	29,403	6.3	-
114	SerPH334	CAH1373104	360	249	22	Clip_A	**K**|INPK	R	D	D	A	G	G	SPH	27,762	8.3	-
115	SerPH36	KAJ3623435	714	245	19	Clip_A Masquerade	**R**|VVGG	H	D	G	D	G	G	SPH	26,090	5.3	-
116	SerPH389	CAH1380739	1324	249	18	Clip_A; Clip_A_Scareface	**P**|GPGP	H	D	Y	D	D	G	SPH	27,371	5.3	-

117	SerPH78	CAH1372746	1180	254	20	Clip_A	**R**∣IKNP	H	D	G	D	G	G	SPH	28,489	5.8	-
118	SerPH544	CAH1363996	313	235	21	Clip_A	**P**∣QKGC	H	D	G	D	G	G	SPH	25,466	9.3	-
119	SerPH203	WJL97983	377	259	16	Clip_B	**K**∣IVHG	H	D	G	D	G	A	SPH	28,414	5.0	-
120	SerPH1005	CAH1363950	321	230	16	Clip_B	**K**∣CGLH	H	D	I	R	G	K	SPH	25,685	7.2	-
121	SerPH312	KAJ3636685	360	252	20	Clip_B	**Q**∣IQGD	H	N	G	D	G	S	SPH	27,612	6.3	-
122	SerPH515	CAH1374578	361	249	16	Clip_C	**R**∣IVGG	Q	D	S	D	G	A	SPH	27,865	6.2	-
123	SerPH589	WJL97984	361	249	16	Clip_C	**R**∣IVGG	R	D	S	D	G	A	SPH	27,672	5.9	-
124	SerPH570	WJL97985	694	233	no	SEA_domain; Fz; LDL (2)	**T**∣VIGE	S	T	F	D	R	R	SPH	26,346	5.8	41-63
125	SerPH364	CAH1369257	2382	253	no	SEA; EGF; LDL (5); SRCR	**R**∣IIGG	S	D	E	D	R	R	SPH	28,155	8.4	250-272

SignalP—Signal peptide; Mm mature—molecular mass of the mature peptidase; pI—isoelectric point of the mature peptidase; TM—transmembrane domain; SerPH—serine peptidase homolog. Regulatory domains: Clip—clip domain (IPR022700), classification by [41]; Fz—Frizzled domain (IPR020067); LDL—Low-Density Lipo-protein receptor type A repeats (IPR002172); SRCR—Scavenger Receptor Cysteine-Rich domain (IPR017448); PAN—Plasminogen-Apple-Nematode domain (IPR003609); SEA— Sperm protein, Enterokinase, and Agrin domain (IPR000082); EGF - laminin/Epidermal Growth Factor-like domain (IPR002049). The amino acids after which the propeptide is cleaved are highlighted in bold.
